# Leveraging heterogeneous data from GHS toxicity annotations, molecular and protein target descriptors and Tox21 assay readouts to predict and rationalise acute toxicity

**DOI:** 10.1186/s13321-019-0356-5

**Published:** 2019-05-31

**Authors:** Chad H. G. Allen, Lewis H. Mervin, Samar Y. Mahmoud, Andreas Bender

**Affiliations:** Department of Chemistry, Centre for Molecular Informatics, Lensfield Road, Cambridge, CB2 1EW UK

**Keywords:** Computational toxicology, Target prediction, Quantitative high-throughput screening, Heterogeneous data

## Abstract

Despite the increasing knowledge in both the chemical and biological domains the assimilation and exploration of heterogeneous datasets, encoding information about the chemical, bioactivity and phenotypic properties of compounds, remains a challenge due to requirement for overlap between chemicals assayed across the spaces. Here, we have constructed a novel dataset, larger than we have used in prior work, comprising 579 acute oral toxic compounds and 1427 non-toxic compounds derived from regulatory GHS information, along with their corresponding molecular and protein target descriptors and qHTS in vitro assay readouts from the Tox21 project. We found no clear association between the results of a FAFDrugs4 toxicophore screen and the acute oral toxicity classifications for our compound set; and a screen using a subset of the ToxAlerts toxicophores was also of limited utility, with only slight enrichment toward the toxic set (odds ratio of 1.48). We then investigated to what degree toxic and non-toxic compounds could be separated in each of the spaces, to compare their potential contribution to further analyses. Using an LDA projection, we found the largest degree of separation using chemical descriptors (Cohen’s *d* of 1.95) and the lowest degree of separation between toxicity classes using qHTS descriptors (Cohen’s *d* of 0.67). To compare the predictivity of the feature spaces for the toxicity endpoint, we next trained Random Forest (RF) acute oral toxicity classifiers on either molecular, protein target and qHTS descriptors. RFs trained on molecular and protein target descriptors were most predictive, with ROC AUC values of 0.80–0.92 and 0.70–0.85, respectively, across three test sets. RFs trained on both chemical and protein target descriptors combined exhibited similar predictive performance to the single-domain models (ROC AUC of 0.80–0.91). Model interpretability was improved by the inclusion of protein target descriptors, which allow the identification of specific targets (e.g. Retinal dehydrogenase) with literature links to toxic modes of action (e.g. oxidative stress). The dataset compiled in this study has been made available for future application.

**Electronic supplementary material:**

The online version of this article (10.1186/s13321-019-0356-5) contains supplementary material, which is available to authorized users.

## Introduction

There are two competing pressures in contemporary chemical risk-assessment. On the one hand, there is increased demand for safety data, for example under the EU’s REACH regulations firms are obliged to submit detailed risk and hazard notifications to the EU for any substance they introduce in significant quantity [1]. On the other hand, there is decreased regulatory and societal acceptance of large-scale traditional in vivo toxicity studies on animals; moreover, on a practical level, covering the vast regions of chemical space requiring toxicity data using the traditional in vivo toxicological techniques would be too time- and resource-intensive to be feasible [2]. The consequence of these pressures is an increased demand for novel methodologies to complement and in some cases replace in vivo studies, including in silico computational toxicology techniques.

Conventional in silico approaches for toxicity prediction include quantitative structure–toxicity relationship modelling, analogous to quantitative structure–activity relationship (QSAR) modelling, whereby machine learning technologies are applied to derive a regression or classification function that maps from chemical structures to their in vivo effect [3]. These approaches have been extensively applied in the domain of toxicity prediction, with some success in cytotoxicity [4, 5], hepatotoxicity [6] and off-target effect prediction [7].

An extension of this approach is the integration of high-throughput in vitro screening data and/or protein target annotations into predictive toxicity modelling. In comparison to toxicity prediction methods that only utilise toxicity structure/structural alerts data alone to provide a prediction [5, 8] these heterogenous approach operate under the hypothesis that chemical, protein target, and phenotypic data domains each contribute partially independent and therefore complementary information about the potential toxic effects of a compound in vivo, and that utilising them in combination may therefore improve the performance of predictive models. Sedykh et al. [9] showed that enhancing a QSAR-style toxicity model with in vitro qHTS data improved its performance. This technique was recently reviewed by Low et al. [10], who, while noting mixed success in its application thus far, expressed optimism that the approach would play a greater role in future of toxicology and drug discovery as the data and expertise required to implement such techniques become more available.

In a previous study [11], we extended this approach by augmenting the dataset of Sedykh et al. with protein target descriptors corresponding to the likelihood of a ligand-target interaction derived from a Bayesian prediction model, representing protein target affinity predictions, and investigated the performance of Random Forest classification models trained to predict binary toxicity classes using successive integration of data domains. We found that, for this data set, inclusion of heterogeneous data domains did indeed generally tend to improve model performance—with a models trained using all three descriptor domains outperforming other combinations of descriptors, with an average correct classification rate (CCR), defined as the mean of sensitivity (true positive rate) and specificity (true negative rate) and also called the balanced accuracy, of 0.82 compared to 0.80 for the next-best model. Models showed the most improvement when chemical descriptors were added to a model which previously lacked them. We also found that the improvement over chemistry-only models was a consequence of more accurate extrapolation of the models’ applicability domain into wider chemical space. However, this study concerned only one small dataset (367 compounds).

Despite the significant increase in data available in the chemical and biological domains, the development of models using several heterogenous data domains still represents a significant challenge. This is due to the collation of a suitable set, given that compounds must simultaneously possess readouts with overlap across several data types, e.g. the structural, bioactivity, phenotypic readout, and toxicological domains (or a subset thereof). In this study, we hence made use of the wealth of toxicity data made available through the Globally Harmonized System of Classification and Labelling (GHS) in order to maximise the overlap of the ToxCast and Tox21 chemical library [12] with a toxicity classification for modelling.

The Globally Harmonized System of Classification and Labelling (GHS) [13] is an international framework for standardising chemical health and safety information. The GHS encompasses a broad spectrum of physical, health and environmental hazards; pertinently for the purposes of this study, this includes the collation of the outcomes of independent toxicity assessments into a set of categories corresponding to the severity of the exhibited toxicity. For three routes of exposure (dermal, inhalation and oral) five categories are defined, each corresponding to a quantitative median lethal dose (LD_50_) interval specified in mg/kg, ppmV or mg/l as appropriate, with the three most severe categories (1–3) necessitating a “toxic” label, category 4 necessitating a “harmful” label, and category 5 requiring no label. The European Chemicals Agency (ECHA), Japan’s National Institute of Technology and Evaluation (JP NITE), New Zealand’s Environmental Protection Authority (NZ EPA) and Safe Work Australia (SWA) provide public access via their websites to governmentally mandated or recommended acute toxicity classifications under the GHS. Further, ECHA publishes the industrial submissions it receives under the requirements of the EU legislation, which include declaring GHS classifications. This data takes the form of the number of notifications received for each GHS hazard category, along with the total number of notifications received. The common classification standards provided by the GHS system enable the collation of acute oral toxicity data from all of these resources with the confidence that the data they hold are mutually commensurate by design. Apart from the ability to look up information on individual compounds, this also represents a valuable means of annotating large compound sets with toxicity labels as performed in this work. The ECHA database has been previously identified as suitable for toxicological data analysis [14], and GHS categories have been used as a framework for defining toxicity thresholds for predictive modelling [15].

In the present study, we annotated 3336 of 8540 standardized chemical structures from the ToxCast and Tox21 chemical library [16] with toxicity classifications derived from regulatory GHS information. We compared the overlap of this compound set with each GHS data source, and the correlations between the sources, and investigated the degree to which GHS-derived toxicity classifications could be discriminated through substructure based screens. We then derived three sets of descriptors for our compounds: molecular descriptors from MOE [17]; in silico-derived protein-target descriptors using an in-house Random Forest ligand-target prediction algorithm [18]; and qHTS activity scores taken from the Tox21 assays disseminated via PubChem [19]. We sought to compare how the GHS toxicity classifications related to these three descriptor sets through analysing the nearest-neighbour distance distributions and linear discrimination analysis projections using the chemical and protein-target descriptors, and the Tox21 qHTS assay data. Finally, we defined and applied three training-test set splits (one random, and two designed to be more challenging) to build and assess the performance of Random Forest classifiers on using these different descriptor sets, and analysed the effect of the inclusion of the different combinations of heterogeneous descriptors on model interpretability.

## Materials and methods

### Dataset collation

For this study, we required compound data for structures, targets, in vitro results, and a toxicity endpoint, which necessitated data collation from multiple sources. To this end, we were able to collate a dataset of 3055 compounds, each of which was annotated with: (1) a chemical structure in SMILES format, from which 2D molecular descriptors were calculated using MOE; (2) qHTS assay results from the Tox21 project published via PubChem; (3) protein target descriptors, representing probabilities of bioactivity against 109 human protein targets; and (4) regulator-derived GHS acute toxicity categorisation for oral, dermal and inhalation exposure routes.

Our starting point was the full ToxCast & Tox21 chemical library [12, 20], as made available for download on the website of the United States’ Environmental Protection Agency [16]. From this, we discarded all compounds which (a) were labelled as “Mixture/Formulation”, “Polymer” or “Macromolecule” in the “Substance_Type” field, or (b) did not possess a CAS registry number (required to lookup GHS categories in regulatory databases). SMILES strings were downloaded from PubChem for any remaining compounds which did not already possess them using the PubChem substance IDs provided or else the CAS registry numbers. This process yielded a set of 8540 compounds for which GHS toxicity annotations could be sought.

### GHS category annotation

Authoritative GHS categorisations were derived from four regulatory classification databases: the harmonized classifications present in ECHA’s Classification and Labelling Inventory [21], the NZ EPA’s Classification and Information Database [22], the GHS classification results published by JP NITE [23], and SWA’s Hazardous Chemicals Information [24]. (Note that while the classifications provided by the NZ EPA are not strictly GHS classifications, the two systems are standardized [25] such that the conversion of the acute toxicity categories to GHS categories is trivial). The authoritative classifications from ECHA, JP NITE and NZ EPA were accessed through the OECD’s eChemPortal service [26] via the CAS registry numbers of compounds; classifications from ECHA and JP NITE were provided directly by eChemPortal, while classifications from NZ EPA were indirectly provided via a link to the compound’s entry on the NZ EPA website. Classification data from SWA were not accessible via eChemPortal, but rather were downloaded directly from the SWA website as a flat file and once again matched to compounds via CAS registry numbers.

There are five acute toxicity categories defined by the GHS, ranging between category 1 (most severe) to category 5 (least severe). However, there is no acute toxicity GHS category directly representing general non-toxicity, since even the least-toxic category represents a closed LD_50_ interval [13]. We have therefore employed the concept of “implied nontoxicity” to ensure sufficient nontoxic compounds were included in the dataset: as GHS classifications are intended to provide a complete and comprehensive overview of a chemical’s hazards, *presence* of a substance in a GHS database and *absence* of an acute toxicity category implies, according to our rationale, nontoxicity. Therefore, we have treated any compound which is present in an authoritative GHS classification database, but which is not categorised under acute toxicity for that administration route, as an implied nontoxic for that administration route. Overall, GHS acute toxicity classifications (including implied nontoxic classifications) from authoritative databases were found for 2770 compounds.

For the remaining 5770 compounds, a GHS categorisation was instead sought using the industrial notifications submitted to ECHA’s Classification and Labelling inventory. Again, eChemPortal was used to connect a CAS registry number to an entry in the ECHA inventory. To minimize the impact of false positives when utilising industrial notification data, no categorisation was applied unless at least 10% of all notifications included that categorisation at either the same or a more severe level (This is the same threshold applied by ECHA to determine whether to advertise a notified hazard on their website and via their data contributions to PubChem). Using this method, 1591 of the total compounds could be associated with a notification-derived classification. Where both a notification-based classification and an authoritative classification were available for the same compound, we preferred the authoritative classification and discarded the notification-based classifications. Overall, 566 additional compound annotations were provided through notification-based classifications, bringing the total number of compounds with a GHS acute toxicity classification to 3336, or 39% of the 8540 compounds for which a classification was sought.

### Structural preprocessing

The last stage of dataset collation was compound filtering and standardization, and the removal of duplicates. The structures as represented by the SMILES strings were standardized using ChemAxon’s Standardizer [27] (the protocol followed was: “remove fragment”, “neutralize”, “remove explicit hydrogens”, “clean 2D”, “mesomerize”, “tautomerize”). Following standardization, the resultant structures were filtered to retain only small organic molecules, by discarding those with no carbon atoms, those containing elements of atomic number 21–32, 36–52 and > 53, and those with molecular weight over 100 Da, leaving 8328 structures.

Finally, a duplicate removal procedure was applied as follows: (1) duplicated structures were identified by converting standardized SMILES to InChIs [28]; (2) all compounds with a duplicated structure but no GHS annotation were discarded; (3) where only one compound in a set of duplicates was also an exact duplicate of its unstandardized structure, that compound alone was retained as the closest representation of the substance for which a GHS annotation was found, and the others discarded (this only occurred for two structures); and (4) any remaining sets of duplicates were discarded. Following this, 7732 substances remained, of which 3060 (40%) possessed GHS annotations.

### Descriptor generation

For each remaining unique compound, molecular descriptors, protein target bioactivity probabilities, and qHTS-derived features were next derived as described in the following.

Firstly, structures were transformed to the major tautomer present at pH 7.4 using ChemAxon’s Calculator [27]. Secondly, a set of 201 2D molecular descriptors were calculated using the Chemical Computing Group’s Molecular Operating Environment (MOE) [17].

Next, to calculate protein target affinity probability profiles we made use of PIDGIN v2 [18], a collection of 3394 target prediction algorithms, trained on over 13 million bioactivity points, with actives (cutoff 10 μM) extracted from ChEMBL [29] and labelled inactives extracted from PubChem [19]. PIDGIN is itself a suite of predictive structure-bioactivity models, providing for each input compound a Platt-Scaled probability of affinity for each target. As with any predictive model its accuracy depends on (in this case) the particular input structure and target class. For that reason, when including its output in further predictive modelling, we chose to only use the output of reasonably reliable models whose applicability domains extended into the dataset at hand in order to minimise the error-carried-forward. To that end, the performance of individual PIDGIN models on the dataset was estimated by measuring the models’ recall on the overlap of the input set and known activities in its training set. Using this estimate, a well-performing model was defined as one which achieved a recall (i.e. proportion of known actives assigned a probability of activity of over 50%) of at least 0.5 on the overlap of the query compounds and the model’s training data. Further, we filtered well-performing models to retain only those with a training set having a mean nearest-neighbour Tanimoto similarity to the 7732 compound set used in this study of over 0.25, calculated using the circular fingerprints utilised by PIDGIN for prediction. These requirements can be considered stringent, as only 109 human target bioactivity models (3% of the total) were retained. This process afforded a set of annotations used as descriptors subsequently. To further ensure that the protein target bioactivity probabilities were as accurate as possible, known bioactivities (i.e. those included in the training data set extracted from ChEMBL) for the compound set on the selected targets were included as probabilities of 1 (i.e. certainty).

Lastly, we assembled qHTS data for our compound set from the Tox21 assay data made publicly available via PubChem. First, all PubChem assay data for all 192 assays listed with “Tox21” as their source were downloaded (accessed on 23 Aug 2018). Next, less relevant assays (i.e. counter-screening assays, autofluorescence assays, and those confirmatory assays for which a summary assay combining its results with counter screens was also available) were discarded, leaving 76 remaining assays (Additional file 1: Table S1). For these assays, the PubChem activity score was used to provide a single qHTS feature summarising the behaviour of a compound against an assay record as a continuous numerical descriptor. Such scores were available for nearly all compounds (7713 out of 7732), but compounds for which no Tox21 scores were available were excluded where necessary. The activity score ranges from 0 to 100, with inactive compounds having a score of 0, active compounds having a score from 40 to 100, and inconclusive compounds having a score in between. The score is provided by the depositor, and the exact way in which the score is calculated depends on the assay, but it is commonly assigned based on potency, efficacy, curve class or a combination of these. Where multiple scores were available for the same structure-assay pairing due to repeated measurements, the median score was used (36% of compounds had at least one repeated measurement). Missing values (11% of all data points) were assumed to be inactive, and assigned a score of 0.

The dataset collated in this study, alongside the code necessary for reproduction of the results obtained, is made publicly available via the Additional files included alongside this article (Additional file 2).

### Binary toxicity classes

For the purposes of defining a binary acute toxicity classification for certain analyses, we considered acute oral toxicity only and took the GHS categories which require a “toxic” (skull and crossbones) pictogram—i.e. categories 1–3—as the toxic class, and those which require no pictogram—i.e. category 5 and implied nontoxicity—as the nontoxic class. Compounds in category 4 (requiring a “harmful” pictogram) were treated as marginal, and disregarded when performing binary analyses. Following this transformation, 2006 compounds were retained of which 579 were classed as toxic and 1427 as nontoxic. Of these, only three compounds lacked Tox21 assay outcomes.

### Exploratory data analysis

The sources’ contributions to the final data were compared by calculating the relative overlap of compounds between the sources, with relative overlap quantified as the intersection over the union. The degree to which the sources were commensurate with one another was considered by calculating the agreement of labels between the GHS acute toxicity categories of the compounds present in both data sources (for the purposes of this analysis, implied nontoxic compounds were allocated to a hypothetical category 6).

The oral bioavailability of the compound set was quantified by considering the fraction of compounds which passed Lipinski’s rule, which was calculated as part of the MOE [17] molecular descriptor set. The druglikeness of the compound set was quantified using DataWarrior’s [30] fragment-based druglikeness score, in which positive values indicate more and negative values less druglike structures.

Visualisation of the coverage of the dataset’s chemical space with GHS annotations was performed using DataWarrior’s Self-Organising Map function [30], using SkelSpheres fingerprints, a Gaussian neighbourhood function, and 100 neurons per axis (i.e. 10,000 neurons in total).

### Toxicophore screening

The structures in the compound set were analysed for the presence of toxicophores using FAFDrugs4 [31] and ToxAlerts [32] in order to investigate the overlap of GHS toxicity classifications with established screens. FAFDrugs4 is an online server designed for filtering compound libraries prior to in silico screening experiments and related modelling studies, which through the “filter undesirable substructure moieties” filtration option provides for the identification of structures containing substructures involved in toxicity which were collated through a manual survey of the literature. For each compound screened by the FAFDrugs4 server, in addition to an inventory of undesirable substructures present, a qualitative screening outcome has been generated (one out of “rejected”, “intermediate” or “accepted”), which depends on the quantity and severity of toxicophores identified.

ToxAlerts is another online platform, available via the Online Chemical Modelling Environment [33], providing structural alerts for the virtual screening of chemical libraries to flag compounds containing toxicity-related substructures. In contrast to FAFDrugs4, ToxAlerts is an open platform allowing user-contributed alerts; however, all alerts must be accompanied by an endpoint and a reference and must be moderated before approval. ToxAlerts therefore contains a wide range of alerts and sources, including sets of alerts derived from literature and from industry. While ToxAlerts contains alerts are all associated with an “endpoint”, certain of these endpoints are generic in nature and do not relate to any defined toxic outcome (e.g. the endpoint “extended functional groups” represents structural features for use in chemical space analysis or as features in further modelling rather than a screening set [34]), and hence the full battery of alerts contained ToxAlerts is not itself suitable for application as a toxicity screen. Rather, a relevant subset of the alerts should be employed. In this study, we used the alerts annotated with the “reactive, unstable, toxic” endpoint, which includes structural alerts defined by ChemDiv, LifeChemicals and Enamine used for their internal compound selection purposes, which was also previously explored in the publication introducing ToxAlerts [32].

### Class separation analyses

We next analysed the various descriptor spaces to determine how informative each was with respect to the GHS toxicity labels. Nearest-neighbour distance analyses were performed as follows: for each compound in our set with a binarized toxicity, the distance to its nearest-neighbour (the most similar compound) in its own class (intra-class) and to its nearest-neighbour in the other class (inter-class) was calculated and the two distributions compared in each space. For the molecular descriptor and the protein target probability spaces, Euclidean distance in MOE and protein target descriptor space was employed to define the nearest-neighbour; for the Tox21 assay scores, due to the sparsity of the data matrix (i.e. a large proportion of zero values), Cosine distance was employed instead. Before calculating distances, the molecular descriptors were centred and scaled (this was unnecessary for the protein target and Tox21 assay descriptor sets, which are each already internally on the same scale). Differences in distribution were tested for significance using a paired *t* test (implemented by SciPy [35]), and effect size measured using Cohen’s *d* (i.e. the difference between two means divided by their pooled standard deviation).

The classes were next analysed to determine which predicted protein targets were more associated with toxic compounds. We used SciPy [35] to perform a paired *t*-test and a Kolmogorov–Smirnov test to determine which target probability descriptors showed a significant difference in distribution between the two classes. We considered a distribution to show a significant difference where the larger of the two *p* values obtained was beneath the Bonferroni-corrected threshold equivalent to *α *= 0.05. Cohen’s *d* was used to quantify the effect size.

We then performed linear discriminant analyses (LDA) as implemented in scikit-learn [36] on the three descriptor spaces to compare their relevance towards the endpoint, by assessing the degree to which a simple linear model might be able to separate the classes in those spaces. An LDA performs feature reduction (to at most one dimension fewer than the number of classes, i.e. to a single dimension in the binary class case) by deriving the linear transformation of the feature space which achieves a maximum separation of the classes; the degree to which the classes separate in the LDA is therefore indicative of the maximal extent to which the classes can be linearly separated in the feature space. The significance of the separation observed under the LDA projection was measured using an unpaired *t*-test, and the effect size using Cohen’s *d*. We examined the weights assigned to the original protein target descriptors by the LDA projection to determine which of these features were found to be more important in linearly separating the classes, and hence which proteins might be involved in causing toxicity in man.

### Predictive modelling

In order to explore the degree to which GHS-derived toxicity labels can be predicted using machine learning models, we defined a predictive modelling set comprising the compounds for which a binary acute oral toxicity class could be defined. Such predictive models are increasingly relevant to chemical hazard assessment, but the robustness of any model is a reliant on the quantity and quality of the data available [37]. For evaluation of predictive models, we defined three test sets. Firstly, we defined a *random* test set, comprising a simple class-stratified random 20% subsample of the modelling set. We then defined two test sets to approximate a more realistic scenario where a predictive model is applied to novel data: a *rare scaffolds* test set (representing novel chemistry) and a *single source* test set (indicating generalisability to novel datasets). The *rare scaffolds* test set comprised all those compounds having a Murcko scaffold [38] present in the modelling set no more than twice (26% of the compounds), the scaffolds having been calculated using RDKit [39]. The *single source* test set comprised a further class-stratified random 20% subsample of the modelling set, but was restricted to include only compounds found in exactly one authoritative GHS data source. In each case, all compounds not included in a test set were used as the corresponding training set.

We applied Random Forest classification models [40] in the predictive modelling analysis, as implemented in scikit-learn [36], because they (a) are non-linear models (complementing the linear transformations performed as part of the exploratory data analysis), (b) are relatively quick to train, even on large numbers of features, (c) require little in the way of feature selection, pre-processing or parameter tuning, and (d) are well-studied algorithms with previous well-performing application in toxicity prediction [11]. For each modelling run, a Random Forest classification model of 200 trees was trained on the relevant training set using scikit-learn default parameters: e.g. *criterion* (the metric for selecting a split) set to “gini”, *max_features* (the number of features considered at each split) set to the square root of the number of features, *max_depth* (the maximum depth of each tree) set to “None” to permit unlimited depth. A fivefold cross-validation routine was employed within the training set to determine the optimum probability threshold (corresponding to the proportion of concurring trees in the ensemble) to be used as the decision boundary, maximising CCR. Each model was then applied to the relevant test set, and its performances assessed in terms of area under the ROC curve and average precision (summarised from the precision-recall curve), along with the sensitivity, specificity and CCR achieved at the probability threshold determined to be optimal from the cross-validation performed within the training set.

Finally, to demonstrate the robustness of the performance statistics computed for these models, and the degree to which they depend upon the random selection of test compounds, the training-test splits were repeated an additional 20 times for those splitting methods where randomness played a role (i.e. the *random* method and the *single source* method). The modelling workflow above was undertaken the performance recorded for each of these repeated splits.

## Results and discussion

### Exploratory data analysis

**Fig. 1:**
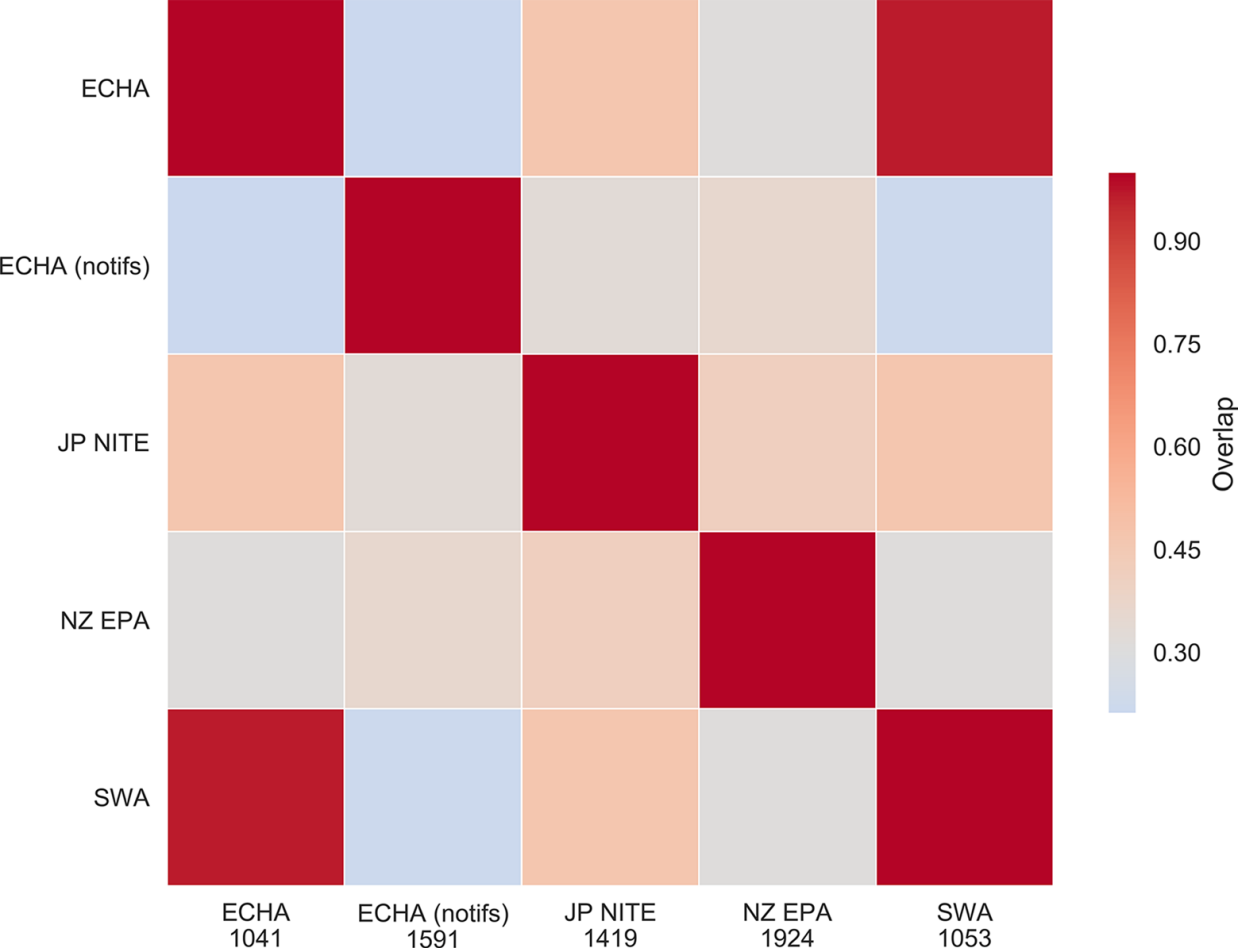
Overlap heatmap for common compounds across the GHS data sources. Overlap here is quantified as intersection over union. Absolute numbers of compounds present in each source are given in the *x* axis. With the exception of the ECHA and SWA data sources (SWA uses ECHA as one of its own sources of classification), the heatmap indicates that other data sources contribute significant quantities of unique data, illustrating the benefit of collating toxicity data from multiple commensurate sources

We firstly compared the coverage of the compound set derived from the ToxCast & Tox21 chemical library across the various sources of GHS classification data. Figure 1 shows the relative pairwise overlap (quantified as the intersection over union) of the presence of the compounds’ CAS registry numbers in the GHS classification databases. We found that most pairs of GHS data sources overlapped by less than 60%, with the exception of the Safe Work Australia and the EU’s harmonized classifications, which were almost wholly overlapped. In particular, the two largest data sources contribute significant novel classifications: the industrial notifications to ECHA has a maximum overlap of 36% with another dataset, and the classifications from the NZ EPA has a maximum overlap of 41%. Hence, while the value of being able to collate commensurate data from multiple sources when assembling such a dataset can be observed, certain data sources are more useful than others in collating a larger dataset.

**Fig. 2:**
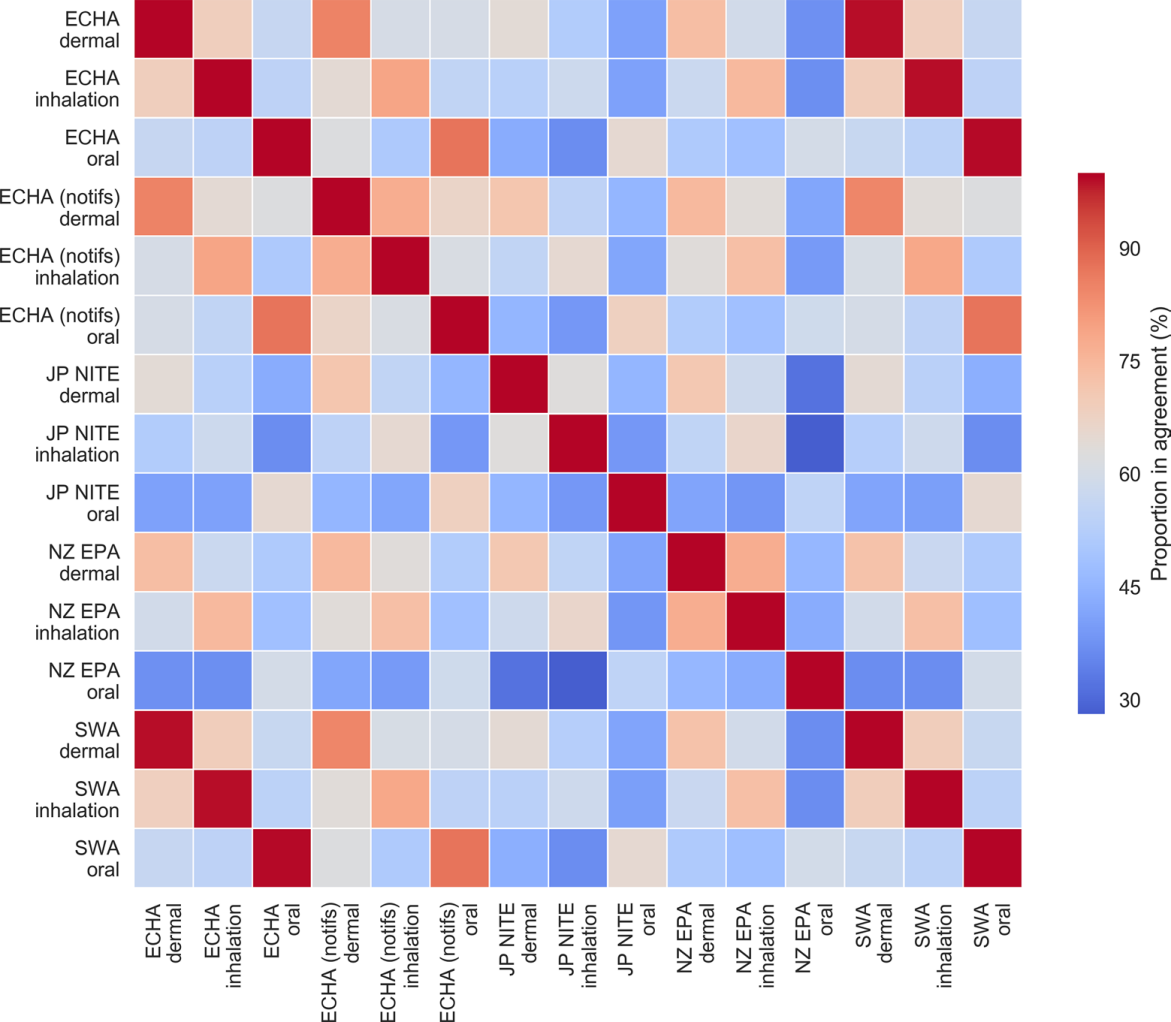
Agreement heatmap between GHS acute toxicity categorisations of common compounds in the GHS data sources. For the purpose of this analysis, implied nontoxic compounds were treated as belonging to a hypothetical category 6. The heatmap indicates that common compounds’ acute toxicity classifications tend to exhibit higher agreement when comparing classifications for the same route of exposure; however, agreement between different routes of exposure are generally substantially lower

In order to test our assumption that GHS data from diverse sources may be pooled, we next compared the classifications obtained for compounds present in multiple data sources. Figure 2 shows the proportion of agreement (i.e. percentage of identical labels) between GHS acute toxicity categories between dermal, inhalation and oral exposure routes and between each data source on common compounds. Two trends are observable. Firstly, that a compound’s acute toxicity category of a given exposure route from a data source exhibits high agreement with its acute toxicity category of the same exposure route from other data sources; for example, 75%, 82% and 80% of compounds exhibited agreement between all oral, dermal and inhalation acute toxicity labels, respectively. This first result is important for the remainder of this study, as the fact that GHS classifications for overlapping compounds exhibit high agreement across regulatory inventories corroborates the notion that GHS classifications are internationally commensurate (as they were intended to be) and can therefore be pooled from disparate sources into a coherent single dataset. Secondly, it can be seen that a compound’s acute toxicity categories across the three exposure routes are poorly correlated across all data sources, with only 35% of compounds having all annotated labels across exposure routes in agreement. The second result simply illustrates that a compound’s toxicity is strongly dependent on its route of exposure, and as some compounds maybe highly toxic via one route but harmless by another the degree to which acute toxicity can be correlated across exposure routes is limited, as has been previously observed. [41] Therefore, rather than attempting to consider acute toxicity independent of route of exposure, in the remainder of this study we considered acute oral toxicity only—as this was the route of exposure with the greatest number of annotations on our compound set.

**Fig. 3:**
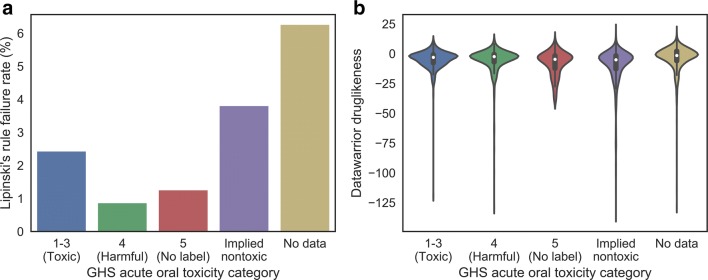
Lipinski’s rule failure rate (**a**) and DataWarrior fragment-based druglikeness score (**b**) for the structures in our compound set. Compounds with an available GHS-derived acute oral toxicity classification (including implied nontoxicity) more frequently pass the Lipinski filter, which may indicate higher bioavailability among those compounds. The distributions (median and inter-quartile range) of druglikeness among the classifications are very similar, though the tail length varies. Hence, we determined that the annotated compounds did not substantially differ with regard to their druglikeness

We next investigated whether there was a difference in druglikeness and Lipinski’s rule failure rate [42] between the GHS classes and the wider ToxCast & Tox21 library, since it was plausible that a GHS classifications might be more often annotated for bioavailable or bioactive compounds. Figure 3 compares the Lipinski’s rule failure rate (serving as a proxy for poor bioavailability) and the fragment-based druglikeness score calculated through DataWarrior [30] across the compounds with and without acute oral toxicity classifications. The results show that no large systematic differences in these properties exist between compounds included or not included in our compound set, nor between the various GHS categories. Although no large differences were found, there was a small (but significant) difference between the proportion of compounds with a GHS classification (including implied nontoxics) and those without passing the Lipinski filter: those with a classification were found to be more likely to pass, at an odds ratio of 2.80 and *p* value of 9.2 × 10^−17^. It is therefore feasible that, while compounds included in this study are no more or less druglike by the DataWarrior metric than those excluded, the compounds for which a GHS-derived acute oral toxicity classification could be applied will tend to be more bioavailable.

The distribution of GHS-derived toxicity classes within the chemical space of the dataset was visualized in through a Self-Organizing Map (Additional file 1: Fig. S1) in which compounds were coloured according to their GHS acute oral toxicity classification. This plot illustrates that GHS categories had been derived for diverse subset of the chemical space described by the wider chemical library, although classifications were not uniformly distributed. The visualisation also reveals a small degree of clustering among the classes.

### Toxicophore analyses

**Fig. 4:**
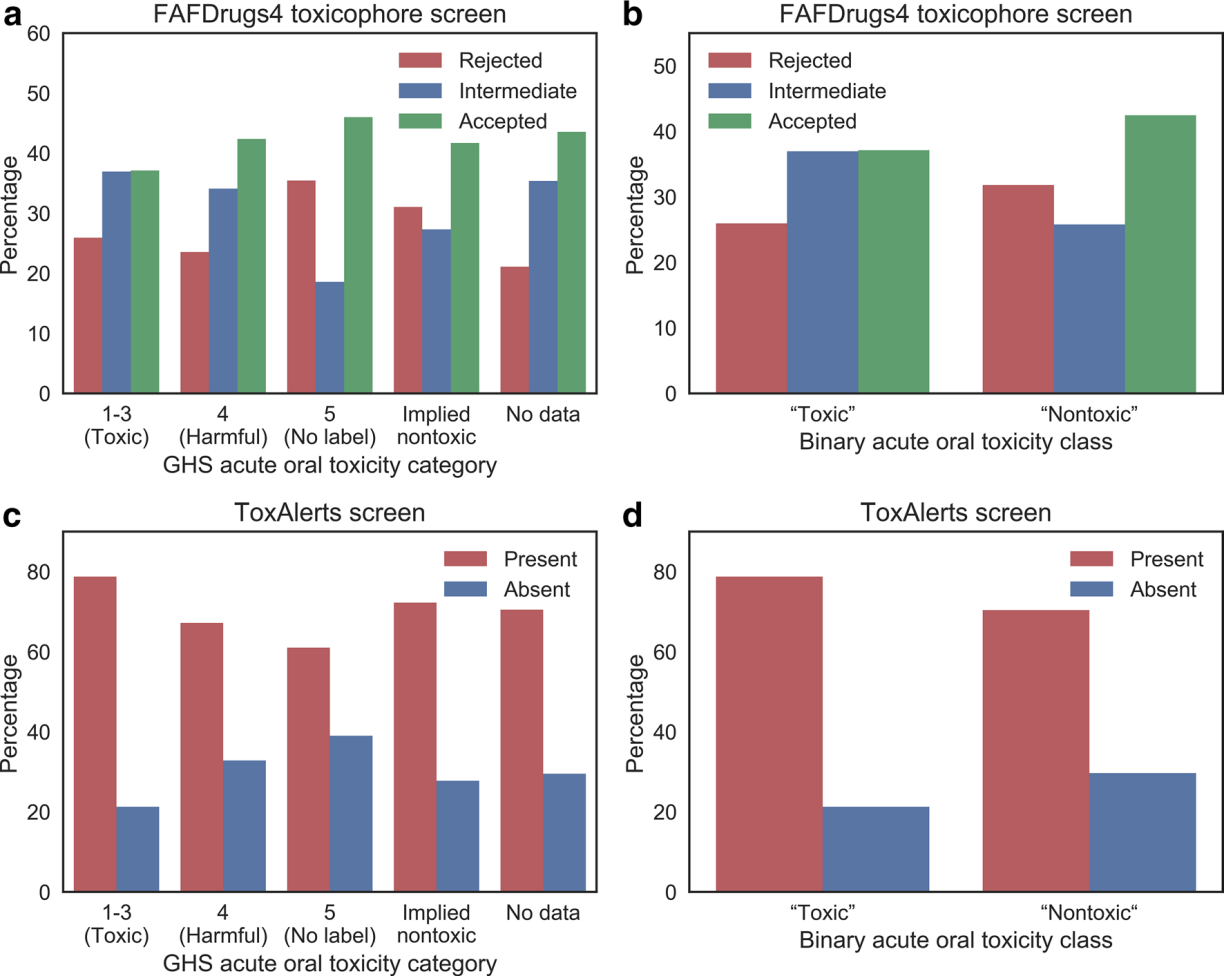
The results of toxicophore screens on the compound set, using the FAFDrugs4 screen (**a**, **b**), and the “reactive, unstable, toxic” endpoint alerts from ToxCast (**c**, **d**). The results of the FAFDrugs4 test are unable to usefully screen for acute oral toxicity as encoded by GHS classes: in B, both rejection (red bar) and acceptance (green bar) is more common in nontoxic compounds. However, there is a weak relationship between these classes and the presence/absence of the “reactive, unstable, toxic” ToxAlerts toxicophores, as illustrated by the higher red bar for the toxic compounds in D

We next examined the degree to which GHS-derived toxicity classifications overlap with existing substructure-based toxicity filters, and sought to identify particular substructures which would be able to screen for these classifications. The results of this toxicophore analysis, broken down by GHS acute oral toxicity class, are given in Fig. 4. It can be seen that there was no clear association between the results of the FAFDrugs4 toxicophore screening test and the GHS acute oral toxicity categories (Fig. 4a). When the compounds are binned into binary toxicity classes (Fig. 4b), a larger fraction of the nontoxic compounds were both accepted and rejected by the screen compared to the toxic compounds, due to the increased number of “intermediate” screening results among the toxic compound set. As an intermediate screening result implies the presence of at least one toxicophore, a conservative approach might reject the intermediate compounds and retain only fully accepted compounds; in which case, it is observed that a significantly higher proportion of nontoxic than toxic compounds were accepted by the FAFDrugs4 screen although the effect size is very small (odds ratio 1.25, *p* value 0.03). However, because of the large proportion of “intermediate” results, measuring the agreement between the FAFDrugs4 screen and the toxicity endpoint is challenging to assess.

To complement FAFDrugs4 screen, we next used the ToxAlerts webserver to screen our compounds as outlined in the Methods section. We identified that ToxAlerts associated with the “reactive, unstable, toxic” endpoint were present in slightly larger proportions of compounds in the more severe acute oral toxicity categories (present in 79%, 67% and 61% of compounds in categories 1–3, 4 and 5, respectively) (Fig. 4c). When the compound set was divided into binary “toxic” and “nontoxic” classes (Fig. 4d), the enrichment of “reactive, unstable, toxic” toxicophores among the compounds annotated as toxic via GHS classifications was measured as having an Odds Ratio of 1.56 with a *p* value of 1.3 × 10^−4^ (representing a significant enrichment, albeit only at a small effect size). This means that while the agreement between FAFDrugs4 alerts and GHS toxicity labels is small, the “reactive, unstable, toxic” substructures from ToxAlerts show a greater prevalence among toxic compounds and therefore may be implicated in contributing towards these compounds’ toxic behaviour.

We next performed an enrichment analysis of the binarized toxic (GHS class 1-3) and compounds compared to nontoxic (class 5 or implied nontoxic) compounds for each toxicophore from ToxAlerts in the “reactive, unstable, toxic” set, to determine which of the individual substructures are associated with the effect. Only those enrichments with a *p* value below the Bonferroni-corrected critical value equivalent to 0.05 (i.e. 0.5/*n*) were considered and the toxicophores satisfying this significance threshold with the largest effect size are given in Table 1. The identified substructures (e.g. double P=S and P=C bonds and thiocarbonyls) represent highly reactive functionalities, consistent with the nature of the category of “reactive, unstable, toxic” endpoints. These reactive moieties or their metabolites may afford covalent modification of biological macromolecules, as the initiating event for many toxicities is the reaction between a xenobiotic electrophile and the nucleophilic regions of important biological peptides and proteins [32, 43]. Hence, in this section we have identified a range of previously-known functional groups associated with toxicity which are present in the GHS toxic dataset.

**Table 1:** Top 5 enriched “reactive, unstable, toxic” endpoint ToxAlerts substructures in the binarized toxic set (GHS acute oral toxicity category of 1–3) versus nontoxic set (acute oral toxicity category of 5 or implied nontoxic)

Toxicophore structure	Alert ID	Description	Odds ratio	*p* value	Source
	TA1000	Double P=S and P=C bonds	35.2	4.7 × 10^−19^	Enamine
	TA975	Thiocarbonyls	27.6	9.6 × 10^−6^	Enamine
	TA880	Gem-Dihalo propane and cyclopropane	25.1	3.1 × 10^−5^	Life chemicals
	TA567	Thioureas	25.1	3.1 × 10^−5^	ChemDiv
	TA885				Life chemicals
	TA1075				Ontario institute for cancer research
	TA914	Nitrosos	16.4	5.0 × 10^−6^	Life chemicals
	TA998				Enamine
	TA1089				Maybridge

Only enrichments with a *p* value below the Bonferroni-corrected cut-off equivalent to α = 0.05 were considered. The remaining significant enrichments were ranked according to their odds-ratio, or effect size. These alerts generally represent reactive functionalities that might be anticipated to afford nonspecific toxicity

While the substructures in the ToxAlerts server labelled with the “reactive, unstable, toxic” endpoint were enriched in compounds classed as “toxic” using GHS acute oral toxicity categories, the size of the effect was small. Indeed, we found that the majority of our toxic and nontoxic compounds contained at least one relevant ToxAlerts alert. These findings corroborate previous findings in the literature concerning the limited utility of relying upon (non-quantitative) structural alerts for accurate toxicity assessment [44], not least due to the propensity for alerts to be present in large proportions of both toxic and nontoxic compounds [45]. Nonetheless, as the GHS is the internationally recognised standard for categorising and communicating chemical hazard generally and acute toxicity specifically, and structural alerts are a widely accepted technique in toxicity screening, we would have anticipated toxicophore analysis to be a more useful means of forecasting its acute oral toxicity categories.

### Class separation analyses

**Fig. 5:**
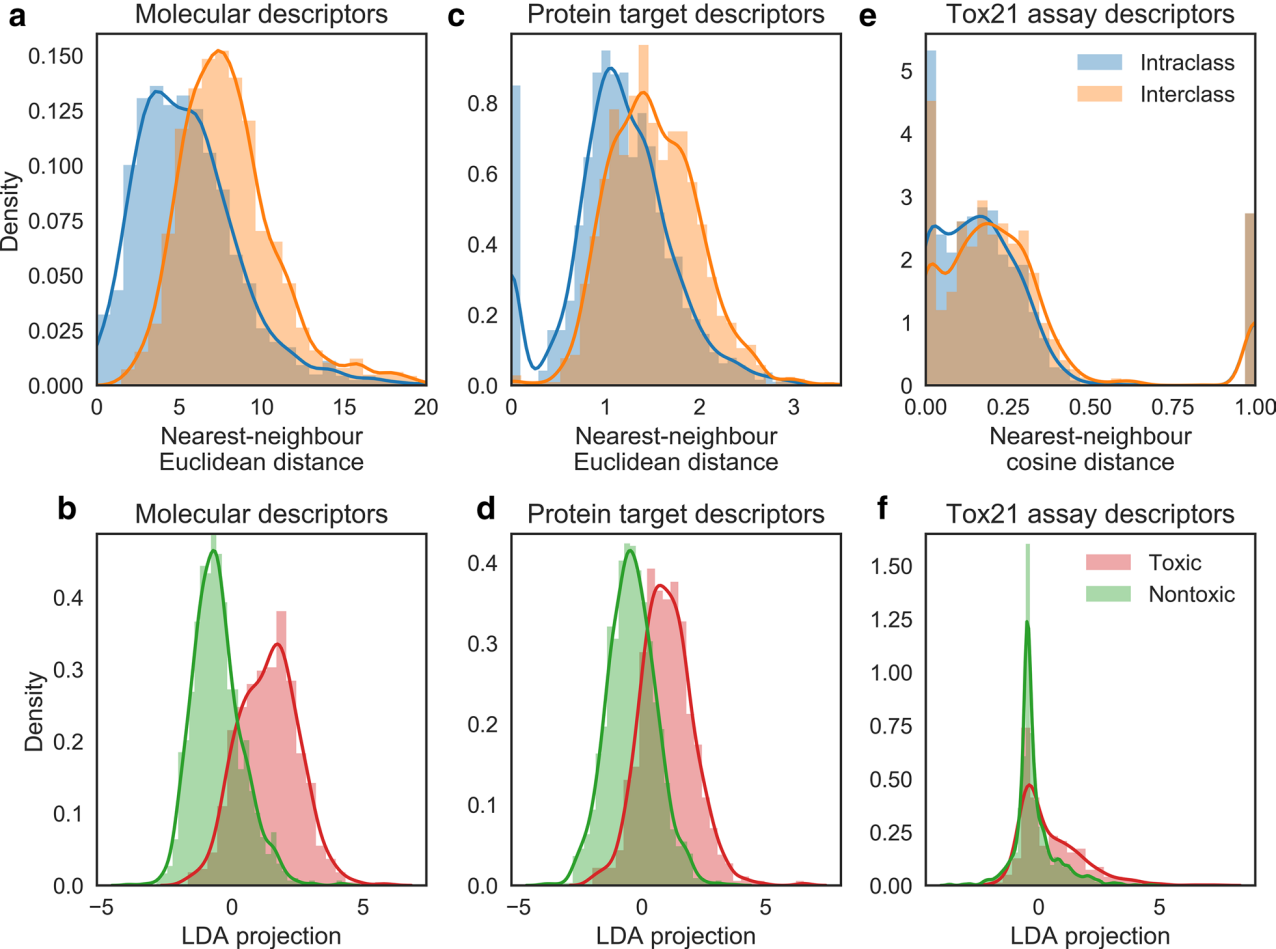
Nearest-neighbour distance distributions for intra-class and inter-class pairs among compounds annotated with a binary toxicity class, and linear discriminant analysis projections on the same data. The molecular descriptor space is used in **a**, **b**, the protein target descriptor space in **c**, **d**, and the in vitro qHTS space in **e**, **f**. Larger differences in nearest-neighbour distributions and LDA projections are evident in the molecular and protein target descriptor spaces than in the in vitro qHTS space

We next analysed the relationship between the binary toxicity classes and the three descriptor domains, to discern to what extent the different toxicity classes can be distinguished in the different descriptor spaces, and thus provide a rationale for implementing and evaluating heterogenous toxicity prediction models. We hence performed nearest-neighbour distance analyses to compare the distributions for inter- and intraclass nearest-neighbours, and LDA projections maximising the interclass distance, in the molecular, protein target, and Tox 21 assay score descriptor spaces (Fig. 5). We shall discuss the nearest-neighbour and LDA plots together, moving from the chemical descriptor space on to the protein target space, and finally onto the Tox21 descriptor space.

In *molecular descriptor space* the nearest-neighbour distance distributions (Fig. 5a) exhibit a large difference in distribution (Cohen’s *d* of 0.72, *p* value of 6.1 × 10^−223^) with interclass nearest-neighbour pairs being further from one another than intraclass pairs, which suggests that the binary toxicity classes exhibit a significant degree of neighbourhood behaviour [46] in chemical space (i.e. small changes in descriptors are associated with no change in class). Likewise, the LDA plot (Fig. 5b) for molecular descriptor space illustrates that a large separation of the classes can be achieved using a linear transformation (Cohen’s *d* of 1.95, *p* value of 2.3 × 10^−253^). Although to a lesser extent, there is also a moderate distinction in *protein target space* in the nearest-neighbour distance distribution (Fig. 5c) (Cohen’s *d* of 0.70, *p* value of 4.4 × 10^−167^) and a comparatively higher degree of separation using LDA (Fig. 5d) (Cohen’s *d* of 1.42, *p* value of 2.8 × 10^−152^). Results from this analysis can be used to determine the protein targets (and hence biological mechanisms) most useful in linearly separating the toxic from non-toxic classes, which is a generally a beneficial feature of utilising interpretable input feature space. Table 2 gives the five most associated protein targets with the toxic set of compounds along with their associated literature evidence to known mechanisms of toxicity (the full list is given in Additional file 1: Table S3). All of the five most associated targets have confirmed links to toxicity, where Retinal dehydrogenase 1 and Nuclear factor erythroid 2-related factor 2 are linked to oxidative stress (ultimately leading to apoptosis) [47, 48] while Nuclear receptor ROR gamma and DNA dC → dU-editing enzyme APOBEC-3F have more direct links to apoptosis and necrosis through their importance in cytokine mediate pathways [49, 50]. The vitamin D3 receptor target can be considered to have a more indirect connection to toxicity, although the disruption its role in Ca^2+^ signalling can be linked to negative regulation of cell proliferation [51]. Table 3 gives the five highest-weighted target probabilities in the LDA projection, where four of those targets have known links to toxicity. For example, the histone deacetylases 5 and 6 are the highest weighted targets, and each are currently exploited in cancer therapies [52, 53]. Additionally, normal function of the Glucagon-like peptide 1 receptor is required for cell proliferation and hence negative regulation can be linked to apoptosis [54]. Kappa-type opioid receptor is also one of the top targets from this analysis but without confirmed literature links to toxicity, which is hence a putative-toxicity related target which we propose could form the basis for future biochemical experiments.

**Table 2:** Protein target descriptors exhibiting a significant difference in distribution between binarized toxic set versus nontoxic set

Uniprot	Protein	Class	Cohen’s *d*	*p* value	Relevant function [associated pathologies]	Refs.
P00352	Retinal dehydrogenase 1	Oxidoreductase	0.48	2.0 × 10^−17^	Metabolism processes and oxidation–reduction process [oxidative stress]	[47]
P51449	Nuclear receptor ROR gamma	Nuclear hormone receptor	0.37	1.4 × 10^−13^	Cytokine-mediated signaling pathways [apoptosis]	[49]
Q16236	Nuclear factor erythroid 2-related factor 2	Transcription factor	0.36	5.5 × 10^−13^	Regulation of cellular redox conditions [oxidative stress]	[48]
Q8IUX4	DNA dC → dU-editing enzyme APOBEC-3F	Hydrolase	0.33	1.5 × 10^−7^	DNA mutators participating in the innate immune system [inducing mutations > apoptosis/necrosis]	[50]
P11473 (pidgin)	Vitamin D3 receptor	Nuclear hormone receptor	0.28	1.0 × 10^−8^	Ca^2+^ signalling [negative regulation of cell proliferation]	[51]

Enrichment effect size was quantified via Cohen’s *d*, and the top 5 targets with the largest effect size indicating more association with the toxic set are shown. Only enrichments with a *p* value below the Bonferroni-corrected threshold equivalent to α = 0.05 were considered. The full table is given in Additional file 1^1^: Table S3. The relevance of these targets to toxicity is explored in the text

**Table 3:** Top 5 highest-weighted human target descriptors in the linear discriminant analysis projection used to discriminate between binarized toxic and nontoxic classes

Uniprot	Protein	Class	Weight (absolute)	Relevant function [Associated pathologies]	Refs.
Q9UQL6	Histone deacetylase 5	Hydrolase	5.45	Chromatin organisation [Negative regulation of cell cycle/apoptosis]	[52]
Q9UBN7	Histone deacetylase 6	Hydrolase	5.19	Chromatin organisation [Negative regulation of cell cycle/apoptosis]	[53]
P41145	Kappa-type opioid receptor		3.02	Central to nerutotranmitter activity i.e.—acetylcholine transport GPCR not the most toxic—could link it to pain [n/a]	
P43220	Glucagon-like peptide 1 receptor		2.60	Activation of cell proliferation [apoptosis]	[54]
P11473	Vitamin D3 receptor	Nuclear hormone receptor	2.20	Ca^2+^ signalling [negative regulation of cell proliferation]	[51]

The relevance of these targets to toxicity is explored in the text

We finally performed the nearest-neighbour and LDA analysis on *Tox21 assay space*, which shows a lower degree of separation on the qHTS descriptors (Fig. 5e, f). The trend of intraclass nearest-neighbours tending to be nearer together than interclass nearest-neighbours was still apparent, but the two distributions were largely overlapping and the effect size was very small (Cohen’s *d* of 0.11, *p* value of 1.4 × 10^−40^). Despite LDA deriving a transformation which achieves the maximal linear separation, still the maxima of the two classes overlapped in the LDA dimension, and the effect size was much less than for the other descriptor spaces (Cohen’s *d* of 0.67, *p* value of 7.5 × 10^−40^). These results suggested that a linear method of class discrimination would have limited applicability using the qHTS values as descriptors. We therefore conclude that the relationship between Tox21 assays and acute oral toxicity is non-trivial, and that the biological mechanisms of toxicity represented in the GHS-derived classifications are not fully captured by the endpoints measured by Tox21; this is partially consistent with the findings of Huang et al. [55], who reported that the performance of models using Tox21 assay data without chemical descriptors to model in vivo toxicity was highly end point dependent, with success only for a subset of endpoints studied.

### Predictive modelling

We next utilized Random Forest classification models in order to evaluate how well different input feature spaces can be used to predict GHS classes computationally. Briefly (for details see methods section), the models were evaluated using three test sets: a *random* test set, a *rare scaffold* test set (containing only compounds having a Murcko scaffold present no more than twice, representing novel chemistry), and a *single source* test set (containing compounds present in only one authoritative GHS data source, indicating generalisability to novel datasets). For each test set, models were trained on all compounds not included in the test set. For each training/test set split, models were generated using each descriptor set in turn. In addition, a fourth class of models were generated using a descriptor set comprising a combination of chemical and protein target descriptors. This resulted in 12 models in total across three test sets and four descriptor sets.

The performances of the resulting models are given in Table 4. Overall, we found that molecular descriptors provided the best predictive performance (best ROC-AUC of 0.92). Next best were protein target descriptors (best ROC-AUC of 0.85), but the models generated using the Tox21 assay descriptors performed poorly (best ROC-AUC of 0.57). As a consequence of these results, we elected to generate a fourth class of model, trained using a combination of molecular and protein target descriptors.

**Table 4:** Summary of Random Forest classifier performances across the three different test sets and the four different combinations of descriptors

Test set	Descriptors	ROC AUC	Average precision	Sensitivity	Specificity	CCR
Random	Molecular	0.92	0.83	0.92	0.78	0.85
	Protein target	0.85	0.71	0.81	0.73	0.77
	Tox21 assay	0.60	0.40	0.47	0.67	0.57
	Molecular and protein target	0.91	0.82	0.85	0.79	0.82
Rare scaffolds	Molecular	0.80	0.68	0.64	0.81	0.72
	Protein target	0.70	0.51	0.70	0.59	0.65
	Tox21 assay	0.57	0.36	0.67	0.43	0.55
	Molecular and protein target	0.80	0.68	0.83	0.63	0.73
Single source	Molecular	0.83	0.65	0.70	0.81	0.75
	Protein target	0.79	0.63	0.76	0.67	0.72
	Tox21 assay	0.61	0.39	0.43	0.73	0.58
	Molecular and protein target	0.85	0.69	0.77	0.76	0.76

Generally, the best performing models were those trained using either molecular descriptors alone or in combination with protein target descriptors. Classifiers found the random test set less challenging to predict than the two more challenging test sets

**Fig. 6:**
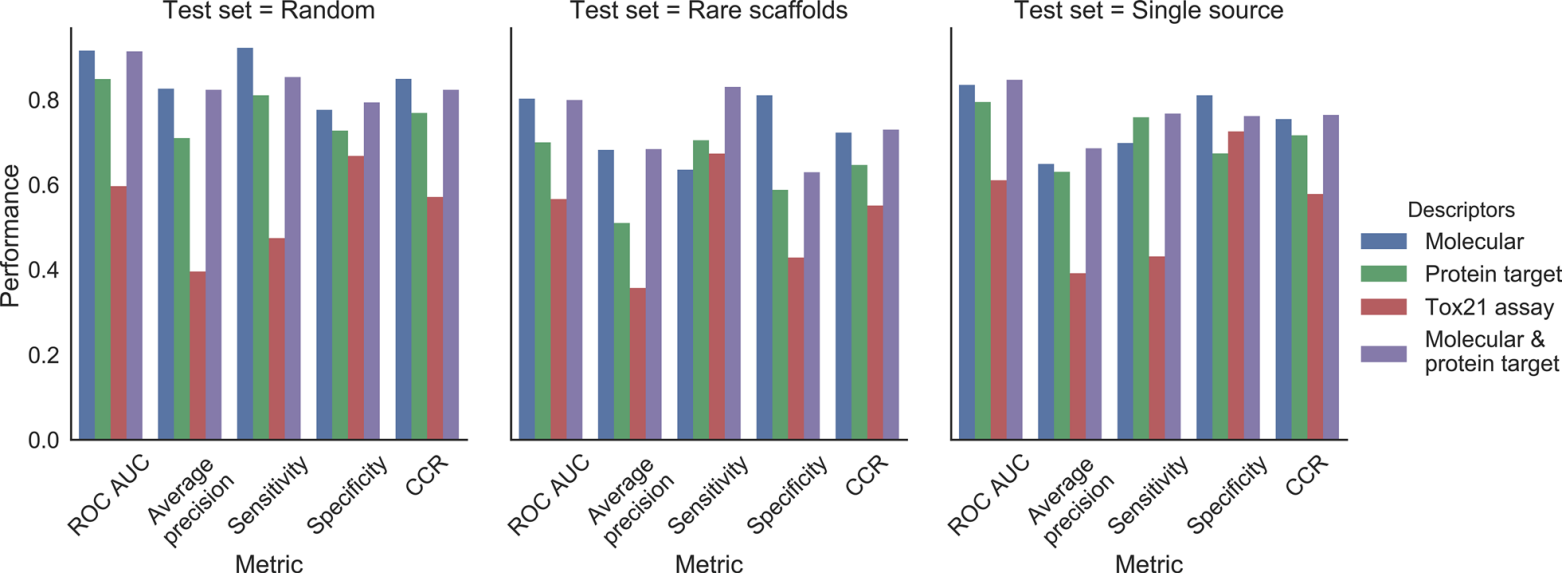
Performance of the four classes of Random Forest classifiers trained on the dataset, quantified by ROC AUC, average precision, and the sensitivity, selectivity and CCR achieved at the optimal prediction threshold, across the three training-test set splits. It can be observed that the best-performing class of models were those utilising molecular descriptors alone or in combination with protein target descriptors. The random test-training split afforded the best-performing models, while performance predicting the toxicity of the rare scaffolds and single source test sets was lower

These results are visualised in Fig. 6 (ROC and precision-recall curves for each model are provided in Additional file 1: Fig. S2 and S3). It can be observed that the models trained using MOE 2D molecular descriptors as features (leftmost, blue bars) exhibited the strongest predictive performance across all three test sets, with a maximum ROC AUC of 0.92 for the random test set and a minimum of 0.80 for the rare scaffolds test set. At the optimal threshold, the model exhibited a CCR of 0.85–0.72 across the three test sets. The strong performance of the model is gratifying in that it illustrates that a relationship can be found between compound chemistry and GHS-derived toxicity classification—in contrast to the relationship between these classifications and structural alerts or toxicophores, which was earlier shown to be weak. The rare scaffold test set provided the greatest challenge to the models, eliciting the weakest performance in the majority of metrics. We attribute to challenging nature of testing compounds with distinct chemistry from model training as encapsulated by their disparate scaffolds. The single source test set also achieved lower validation metrics than the random test set, with a reduction in the CCR from 0.85 to 0.75 respectively, which is only 0.03 higher than the performance obtained on the rare scaffold test set. Hence, taken together, our findings show that physico-chemical descriptors are a powerful feature set for learning the GHS toxicity classifications. This is despite their lack of molecular context, but we attribute this in part to their relevance to ADME mechanisms.

The models trained on protein target probabilities achieved good performance overall across the test sets (centre-left, green bars in Fig. 6), with ROC AUCs between 0.85 and 0.70, average precisions between 0.71 and 0.51, and CCRs (at the optimum thresholds) between 0.77 and 0.65 across the test sets. Despite this, ROC AUC, average precision and CCR were each always lower than the equivalent value for the model built using molecular descriptors for every test set. This result corroborates the finding of our prior work, [11] in which we found an average CCR drop of 0.11 across 100 training-test splits comparing Random Forest models trained on molecular descriptors to those trained on protein target descriptors.

The predictive performances of the models trained on molecular descriptors and the models trained on protein-target descriptors were further analysed by considering only those compounds which were classified differently by the two models in any of the training-test set splits, using their optimum thresholds. A full list of these compounds is provided in Additional file 3, and summarised in Additional file 1: Table S4. It is observed that for such compounds, models trained using molecular descriptors are generally more likely to make the correct prediction. However, this was not the case for toxic compounds in the rare scaffolds test set (for which the protein-target descriptor-trained model made the correct prediction 61.2% of the time) and toxic compounds in the single source test set (for which the protein-target descriptor-trained model made the correct prediction 62.1% of the time). Where the two models disagreed on nontoxic compounds in these test sets, the molecular descriptor-trained models more frequently made the correct classification (78.3% of cases for the rare scaffolds set and 74.7% of cases for the single source set). For compounds within the random test set where the two classifiers disagreed, the molecular descriptor-trained model performed better on both toxic and nontoxic compounds (88.2% and 60.6% correct, respectively). However, these compounds were the only ones for which the protein-target descriptor-trained model made a larger proportion of correct predictions on nontoxic than toxic compounds—though it was out-performed by the very strong molecular descriptor-trained model (ROC AUC of 0.92) in each case.

In contrast to the aforementioned models, the models built using data derived from in vitro data from the Tox21 project exhibited comparatively poor predictivity across the test sets (centre-right, red bars in Fig. 6), with ROC AUCs varying from 0.61 to 0.57, average precision scores varying from 0.40 to 0.36, and CCRs at the optimum threshold varying from 0.57 to 0.55. These results indicate that the model performed only slightly better than a random classifier, which is particularly evident in the ROC plots in Additional file 1: Fig. S2. Though this is a disappointing result, it reflects the smaller separations observed between classes seen in earlier exploratory analyses (Fig. 5). Indeed, there has been mixed success in the literature when attempting to use high-throughput screening assays to predict in vivo toxicity. In order to link our work to existing studies, we consider the review of Thomas et al. [56] who found in their comprehensive review of chemicals and assays provided under ToxCast phase I (a closely related endeavour to the Tox21 project) that those assays have “limited applicability for predicting in vivo chemical hazards using standard statistical classification methods.” In contrast, the study of Huang et al. [55] which reported variable success utilizing Tox21 assays alone to predict in vivo endpoints found that “combing structure and activity data resulted in better models than those built with structure or activity data alone” for most of the endpoints they studied. The difference in results may be surprising given the overlap between the compound set and the Tox21 assays, however this previous study differed from ours in that they made use of a Self-Organizing Map-based approach in contrast to our Random Forest, and different toxicity endpoints were considered. It is reasonable to conclude that the success of utilizing the Tox21 assay data depends on optimizing the analysis for that purpose, rather than comparing their performance as simply another class of descriptor in a study such as this. Moreover, the such qHTS assay data has applications beyond predictive modelling, including deriving putative mechanisms for adverse events [57].

We also generated a fourth class of models using both molecular and protein target descriptor sets. The predictive performance of this model class is also given in Table 4 and illustrated by the far right, purple bars in Fig. 6. Performance measured by ROC AUC, average precision and CCR for this class of model tended to be very similar to the molecular descriptors-only model class across the three test sets (differing by at most 0.03), though sensitivity and specificity showed more variation (differing by at most 0.19 and 0.18, respectively). The maximum discrepancy occurred for the rare scaffold set, for which the combination model exhibited a sensitivity 0.19 higher than the molecular descriptor only model, and a selectivity 0.18 lower. Overall, however, the CCR remained comparable.

Finally, to examine the dependence of these results on the randomness in the division of test and training instances, the training–testing routines were performed as above for 40 further models: 20 using further class-stratified random splits, and 20 using further single-source splits (in which a class-stratified random subsample of single-source compounds are selected for the test set). Due to the design of the rare scaffold set, it was not possible to repeat this split to generate multiple test sets. The mean and sample standard deviation (SD) of the performance metrics for multiple random-split models are given in Additional file 1: Table S5, and for the multiple single-source splits in Additional file 1: Table S6. These results indicate that the performance of the models analysed in detail elsewhere in this study are broadly representative of other random splits that might have been chosen. In particular, the same relative performance trends for the models trained both on varying descriptor sets and varying training sets are observed in the models analysed in detail and in the summary statistics. One key observation is that, on average, models trained using only molecular descriptors and models trained using both molecular and protein-target descriptors perform very similarly on average for both random splits [mean ROC AUC of 0.941 and 0.913 (SD of 0.013 in each case), respectively] and single source splits [mean ROC AUC of 0.851 and 0.852 (SD of 0.013 in each case again), respectively]. The average performances of models trained using the Tox21 assay descriptors remained low, with the best average performance seen in the random test sets producing a ROC AUC of 0.619 (SD of 0.034) and a CCR of 0.619 (SD of 0.029).

The natural question arising from these results is whether the additional complexity introduced by integrating protein target bioactivity probabilities into the molecular descriptor set is worthwhile, given that the effect on predictive performance is minimal (or marginally detrimental, for certain metrics). Table 5 compares the most important features, determined according to the expected fraction of decisions utilising that feature, in the two most predictive models studied in detail here: the model using chemical descriptors alone and the model using chemical and protein target descriptors in combination, both built using the random test set split. This table allows one potentially benefit of including protein target descriptors to be examined: the potential for an increase in model interpretability afforded by including protein target descriptors.

**Table 5:** Highest-importance features in the two Random Forest classifiers with the highest ROC AUC scores, i.e. those generated using the random test-training set split using (a) molecular descriptors only and (b) both molecular and protein target descriptors

Classifier	Highest importance molecular descriptors	Highest importance protein target descriptors
(a) Random test set, molecular descriptors	*a_nN:* Number of nitrogen atoms *Q_RPC*-: Relative negative partial charge *a_ICM*: Atom information content (mean) *h_pavgQ:* Average total charge sum across protonation states at pH 7 *GCUT_PEOE_0:* First GCUT descriptor calculated from the eigenvalues of a modified graph distance adjacency matrix where the diagonal takes the values of the partial charges	*n/a*
(b) Random test set, molecular and protein target descriptors	*Q_RPC*-: Relative negative partial charge *a_nN:* Number of nitrogen atoms *GCUT_SLOGP_0:* First GCUT descriptor calculated using atomic contributions to logP instead of partial charge *bpol:* Sum of the absolute value of the difference between atomic polarizabilities of all bonded atoms in the molecule *chi1v_C:* Carbon valence connectivity index (order 1)	*P18031:* Tyrosine-protein phosphatase non-receptor type 1 *P51449:* Nuclear receptor ROR-gamma *P00352:* Retinal dehydrogenase 1 *P23219:* Prostaglandin G/H synthase 1 *P11473:* Vitamin D3 receptor

The table illustrates the difference in interpretability between the two classes of descriptors, since molecular descriptors may be either be too broad to interpret or nontrivial to understand, while protein target descriptors provide a specific biological hypothesis which can be subsequently tested to validate a mechanism of action

While the MOE-generated molecular descriptors are evidently highly predictive for the data set employed in the present study, and therefore of great utility where the priority is simply the accuracy of the model, it’s not immediately obvious what some of the more esoteric descriptors represent (e.g. connectivity indices), nor how they might relate to acute oral toxicity. This means that generating any further insight from these models is a challenge. In contrast, as discussed above, protein target descriptors can be more readily interpreted through literature validation. Two of the most of important protein targets were not previously identified through the exploration of descriptor spaces, namely Tyrosine-protein phosphatase non-receptor type 1, a kinase linked with cytoskeletal machinery and interferon pathways (GO:0060338) [58], and Prostaglandin G/H synthase 1 which has links to cellular stress events [59]. Three of the five were previously identified in the aforementioned nearest-neighbour and LDA analysis (Nuclear receptor ROR-gamma, Retinal dehydrogenase 1 and Vitamin D3 receptor), which illustrates that the RF algorithm is able to identify these target descriptors as an important feature to separated toxicity classes in addition to its predictive functionality. The targets identified in this work, via the feature importance and descriptor distribution analysis, share overlap with the cytotoxic target enrichment analysis (Fisher Test) presented in the complete list included in Mervin et al. [5], namely DNA dC → dU-editing enzyme APOBEC-3F, Glucagon-like peptide 1 receptor and Tyrosine-protein phosphatase non-receptor type 1, in addition to the histone deacetylases 5 and 6, as identified in both Mervin et al. and Liggi et al. [4, 5].

## Conclusions

We have presented the collation of a novel dataset, not previously analysed, which comprised acute toxicity labels (derived from GHS data made available by regulatory authorities), chemical structures and qHTS results from Tox21. Molecular descriptors were derived from the chemical structures, compounds were annotated protein target descriptors using in silico target prediction, and the qHTS results were summarised into a descriptor set.

In our exploration of the descriptor dataset, we found those compounds with a GHS-derived acute oral toxicity labels were not substantially more or less druglike than the full ToxCast & Tox21 chemical library. We found that acute oral toxicity, as encoded by the GHS system, was not well aligned with the FAFDrugs4 toxicophore-based screen. In contrast, a subset of the toxicophores from the ToxAlerts server exhibited a modest relationship with GHS-encoded acute oral toxicity. We therefore conclude that toxicophore-based screens cannot alone discern the acute toxicity encoded within the GHS. We found that the acute oral toxicity classes derived from GHS data were partially linearly separable in chemical and protein-target space, as illustrated using nearest-neighbour distance distributions and linear discriminant analyses. Little separation was observed in the Tox21 descriptor space, in agreement with our previous studies.

Predictive models could be created by training Random Forest models on the dataset using molecular descriptors and protein target bioactivity probabilities as input features, with the model trained on the molecular descriptors outperforming that trained on the bioactivities (CCRs of 0.85–0.72 compared to 0.77–0.65). However, the qHTS data from the Tox21 assays could not be successfully employed in GHS class prediction, with the Random Forest model trained using these as features exhibiting a CCR little better than a random guess. We conclude from this that the endpoints captured by the Tox21 project may not be relevant to the acute in vivo toxicity encoded by the GHS classifications, or else that the relationship between the two may not be captured by the simplistic application of the Tox21 assay results as features in a supervised machine learning algorithm as expected from the separations observed in the three spaces.

A combined model trained on both chemical descriptors and protein target bioactivity descriptors had similar predictive performance to that trained on chemical descriptors only. This result was confirmed by measuring the average performance of 40 further models trained and tested on repeated randomised splits. We suggested that, given that the performance of the combined model was comparable with the chemistry-only model, such a combined model may be primarily useful due to the increased interpretability of the feature importance extractable from the model. The five most relevant protein target descriptors had links to toxicity in the literature. Further work on this dataset may focus on exploring the degree to which this interpretability can be harnessed toward interpretability and mode-of-action hypothesis generation on a compound-by compound basis. We have made the dataset publicly available to this end.

## Additional files

**Additional file 1.** Supplementary information.

**Additional file 2.** Research code and data.

**Additional file 3.** List of compounds for which molecular and protein target models disagree.

## Data Availability

The molecular and protein target descriptors, and the regulator-derived GHS toxicity annotations used in this article are available in the Additional files. The Tox21 assay data is available on PubChem, via the assay IDs listed in Additional File 1: Table S1.

## Abbreviations

CCR: (class-balanced) correct classification rate, i.e. the mean of sensitivity and specificity
ECHA: European Chemicals Agency
JP NITE: Japan’s National Institution of Technology and Evaluation
LDA: linear discriminant analysis
NZ EPA: New Zealand’s Environmental Protection Authority
ROC: receiver operating characteristic curve
ROC AUC: area under the receiver operating characteristic curve
SWA: Safe Work Australia
