# Supplementary material for: Leveraging heterogeneous data from GHS toxicity annotations, molecular and protein target descriptors and Tox21 assay readouts to predict and rationalise acute toxicity
Source: J Cheminform. 2019 May 31;11:36. doi: 10.1186/s13321-019-0356-5 (PMC6544914; doi:10.1186/s13321-019-0356-5)
Supplement: Supplementary file 1 — Additional file 1. Supplementary information. [file 13321_2019_356_MOESM1_ESM.docx]

Leveraging heterogeneous data from GHS toxicity annotations, molecular and protein target descriptors and Tox21 assay readouts to predict and rationalise acute toxicity:
Supplementary information

Chad H. G. Allen, Lewis H. Mervin, Samar Y. Mahmoud, and Andreas Bender


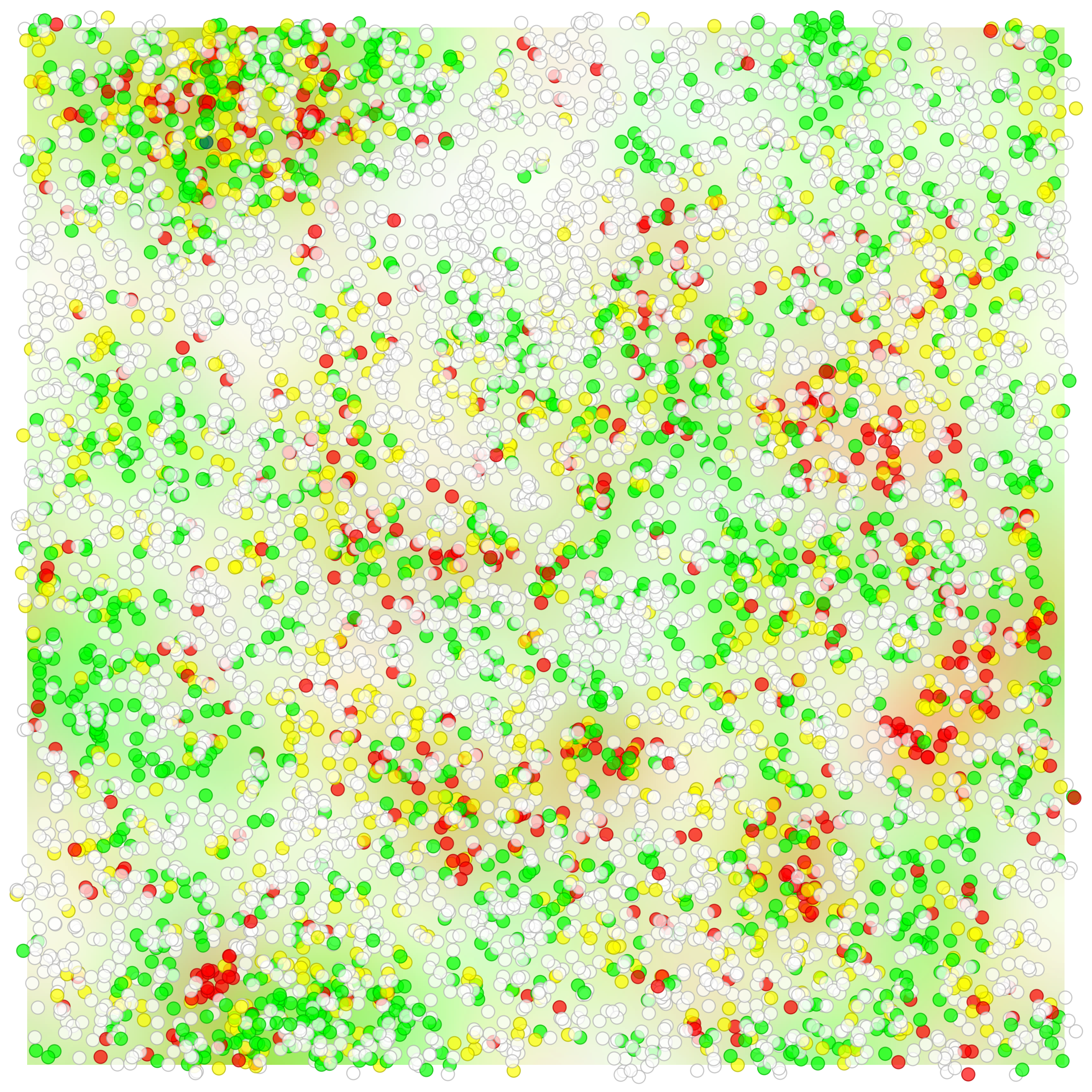


**Figure S1**. Self-organising map trained on SkelSphere fingerprints calculated by DataWarrior from the compound set. Compounds with an acute oral toxicity GHS class of 1-3 (“toxic”) are plotted in red, those with an acute oral toxicity class of 4 (“harmful”) in yellow, and those with a class of 5 or which are implied nontoxic in green. Those which could not be classed are plotted in white. This plot illustrates the degree to which GHS-derived acute oral toxicity classifications cover the regions of chemical space spanned by the compounds in the library analysed in this study. The self-organising map also displays some degree of class-separation in chemical space (e.g. regions with fewer toxic compounds in the centre-left and top-right, and several clusters of toxic/harmful compounds to the centre-right of the plot).


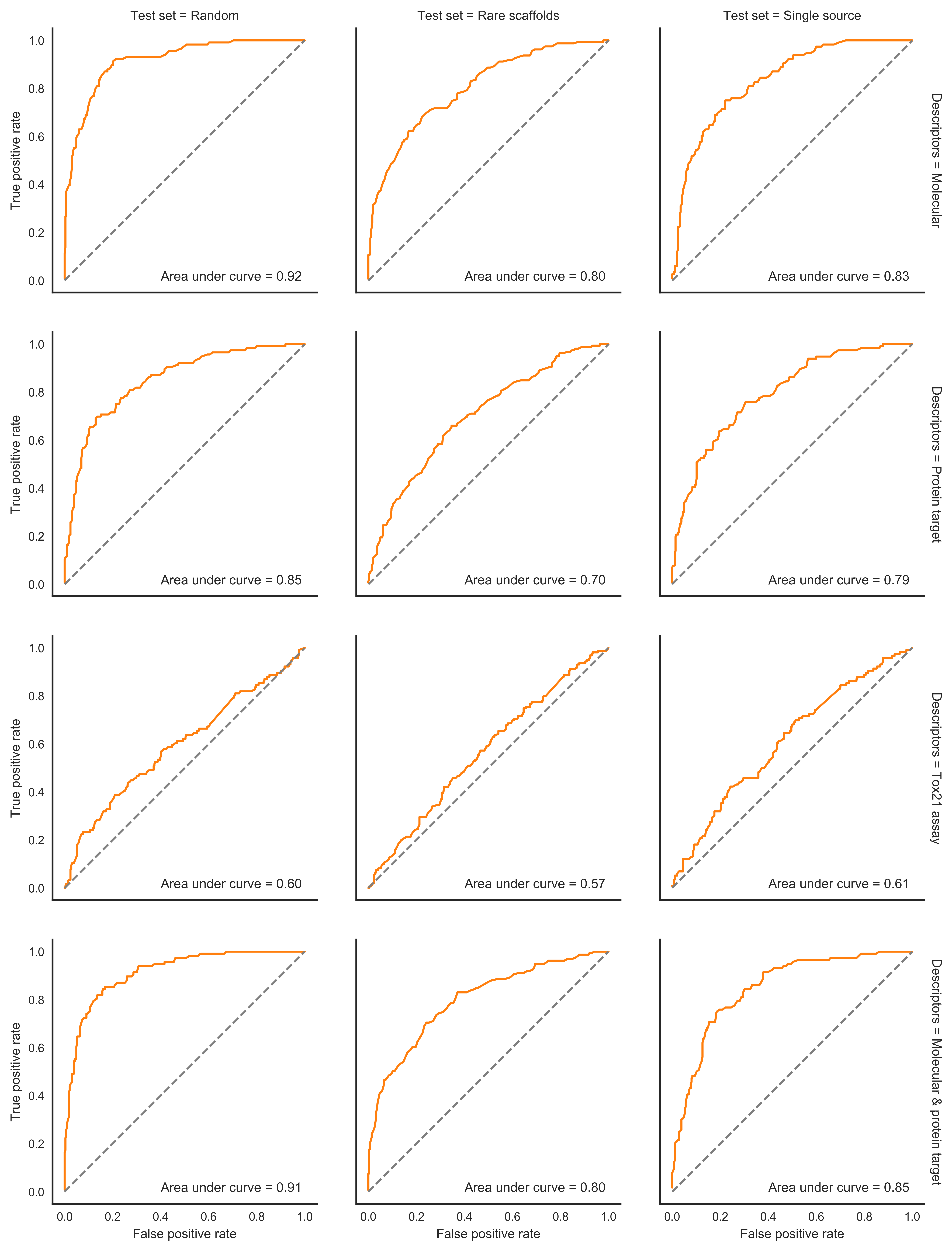


**Figure S2**. ROC curves (true positive rate against false positive rate for varying classification threshold) for each random forest model trained in the study, arranged by test set (columns) and descriptor set (rows). The strongest performing models were those trained using molecular descriptors (top) and molecular descriptors and protein target descriptors combined (bottom). Performance on the random test set was generally strongest, and on the rare scaffold set generally poorest. The ROC curves for the models trained using Tox21 assay descriptors (second row from bottom) do not rise far above the diagonal, indicating predictive performance not substantially better than random.


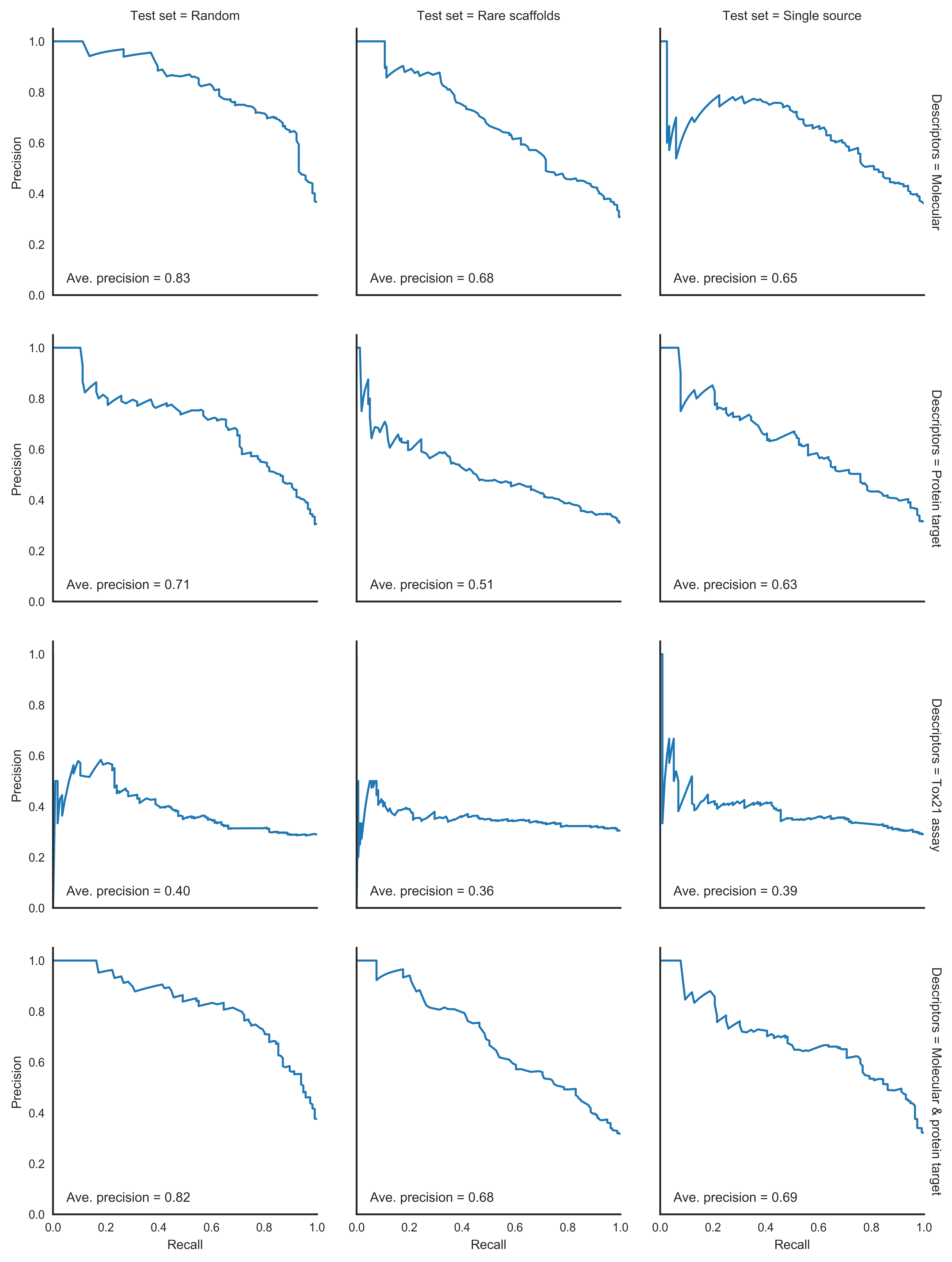


**Figure S3**. Precision-recall curves for each random forest model trained in the study, arranged by test set (columns) and descriptor set (rows). As with the ROC curves in Figure S2, models trained using molecular descriptors (top) and molecular descriptors and protein target descriptors combined (bottom) showed the strongest performance, and the performance of the models trained using the Tox21 assay descriptors was poorest.

**Table S1.** PubChem Assays used to provide qHTS-derived descriptors.

| **PubChem AID** | **Assay name** |
| --- | --- |
| 1159518 | qHTS assay to identify small molecule agonists of the NFkB signaling pathway: Summary |
| 1159519 | qHTS assay to identify small molecule agonists of the endoplasmic reticulum stress response signaling pathway: Summary |
| 1159523 | qHTS assay to identify small molecule antagonists of the retinoid-related orphan receptor gamma (ROR-gamma) signaling pathway: Summary |
| 1159528 | qHTS assay to identify small molecule agonists of the AP-1 signaling pathway: Summary |
| 1159531 | qHTS assay to identify small molecule agonists of the RXR signaling pathway: Summary |
| 1159555 | qHTS assay to identify small molecule antagonists of the retinoic acid receptor (RAR) signaling pathway: Summary |
| 1224867 | qHTS RealTime-Glo MT Cell Viability Assay in HepG2 cells - 24 hour |
| 1224868 | qHTS RealTime-Glo MT Cell Viability Assay in HEK293 cells - 32 hour |
| 1224869 | A CellTox Green Cytotoxicity Assay to monitor cytotoxicity in HEK293 cells - 0 hour |
| 1224870 | qHTS RealTime-Glo MT Cell Viability Assay in HepG2 cells - 40 hour |
| 1224871 | A CellTox Green Cytotoxicity Assay to monitor cytotoxicity in HEK293 cells - 40 hour |
| 1224872 | qHTS RealTime-Glo MT Cell Viability Assay in HEK293 cells - 16 hour |
| 1224873 | qHTS RealTime-Glo MT Cell Viability Assay in HepG2 cells - 8 hour |
| 1224874 | qHTS RealTime-Glo MT Cell Viability Assay in HEK293 cells - 40 hour |
| 1224875 | A CellTox Green Cytotoxicity Assay to monitor cytotoxicity in HEK293 cells - 24 hour |
| 1224876 | A CellTox Green Cytotoxicity Assay to monitor cytotoxicity in HepG2 cells - 16 hour |
| 1224877 | qHTS RealTime-Glo MT Cell Viability Assay in HepG2 cells - 32 hour |
| 1224878 | A CellTox Green Cytotoxicity Assay to monitor cytotoxicity in HepG2 cells - 24 hour |
| 1224879 | A CellTox Green Cytotoxicity Assay to monitor cytotoxicity in HepG2 cells - 40 hour |
| 1224880 | qHTS RealTime-Glo MT Cell Viability Assay in HEK293 cells - 0 hour |
| 1224881 | A CellTox Green Cytotoxicity Assay to monitor cytotoxicity in HEK293 cells - 32 hour |
| 1224882 | A CellTox Green Cytotoxicity Assay to monitor cytotoxicity in HepG2 cells - 0 hour |
| 1224883 | A CellTox Green Cytotoxicity Assay to monitor cytotoxicity in HepG2 cells - 32 hour |
| 1224884 | A CellTox Green Cytotoxicity Assay to monitor cytotoxicity in HEK293 cells - 8 hour |
| 1224885 | qHTS RealTime-Glo MT Cell Viability Assay in HepG2 cells - 16 hour |
| 1224886 | qHTS RealTime-Glo MT Cell Viability Assay in HEK293 cells - 24 hour |
| 1224887 | qHTS RealTime-Glo MT Cell Viability Assay in HEK293 cells - 8 hour |
| 1224888 | A CellTox Green Cytotoxicity Assay to monitor cytotoxicity in HEK293 cells - 16 hour |
| 1224889 | qHTS RealTime-Glo MT Cell Viability Assay in HepG2 cells - 0 hour |
| 1224890 | A CellTox Green Cytotoxicity Assay to monitor cytotoxicity in HepG2 cells - 8 hour |
| 1224892 | qHTS assay to identify small molecule agonists of the constitutive androstane receptor (CAR) signaling pathway: Summary |
| 1224893 | qHTS assay to identify small molecule antagonists of the constitutive androstane receptor (CAR) signaling pathway: Summary |
| 1224894 | qHTS assay to identify small molecule agonists of the hypoxia (HIF-1) signaling pathway: Summary |
| 1224895 | qHTS assay to identify small molecule agonists of the thyroid stimulating hormone receptor (TSHR) signaling pathway: Summary |
| 1224896 | qHTS assay to identify small molecule agonists of H2AX: Summary |
| 1259247 | qHTS assay to identify small molecule antagonists of the androgen receptor (AR) signaling pathway using the MDA cell line in the presence of 0.5 nM R1881: Summary |
| 1259248 | qHTS assay to identify small molecule antagonists of the estrogen receptor alpha (ER-alpha) signaling pathway using the BG1 cell line in the presence of 0.1 nM 17-beta-estradiol: Summary |
| 1259387 | qHTS assay to identify small molecule agonists of the androgen receptor (AR) signaling pathway in the presence of an antagonist: Summary |
| 1259388 | qHTS assay to identify small molecule HDAC inhibitors: Summary |
| 1259390 | qHTS assay to identify small molecule agonists of the sonic hedgehog signaling (Shh) pathway: Summary |
| 1259391 | qHTS assay to identify small molecule agonists of the estrogen receptor alpha (ER-alpha) signaling pathway in the presence of an antagonist: Summary |
| 1259392 | qHTS assay to identify small molecule antagonists of the sonic hedgehog signaling (Shh) pathway: Summary |
| 1259394 | qHTS assay to identify small molecule agonists of the estrogen receptor beta (ER-beta) signaling pathway: Summary |
| 1259395 | qHTS assay to identify small molecule antagonists of the thyroid stimulating hormone receptor (TSHR) signaling pathway: Summary |
| 1259396 | qHTS assay to identify small molecule antagonists of the estrogen receptor beta (ER-beta) signaling pathway: Summary |
| 1259401 | qHTS assay to identify small molecule antagonists of the estrogen related receptor signaling pathway with the pleiotropic PPARgamma coactivator (PGC) from Tox21 10K library: Summary |
| 1259402 | qHTS assay to identify small molecule agonists of the estrogen related receptor signaling pathway with the pleiotropic PPARgamma coactivator (PGC) from Tox21 10K library: Summary |
| 1259403 | qHTS assay to identify small molecule antagonists of the estrogen related receptor (ERR) signaling pathway from Tox21 library: Summary |
| 1259404 | qHTS assay to identify small molecule agonists of the estrogen related receptor (ERR) signaling pathway from Tox21 library: Summary |
| 720516 | qHTS assay for small molecules that induce genotoxicity in human embryonic kidney cells expressing luciferase-tagged ATAD5: Summary |
| 720552 | qHTS assay for small molecule agonists of the p53 signaling pathway: Summary |
| 720637 | qHTS assay for small molecule disruptors of the mitochondrial membrane potential: Summary |
| 720719 | qHTS assay to identify small molecule agonists of the glucocorticoid receptor (GR) signaling pathway: Summary |
| 720725 | qHTS assay to identify small molecule antagonists of the glucocorticoid receptor (GR) signaling pathway: Summary |
| 743012 | qHTS assay for identifying genotoxic compounds that show differential cytotoxicity against isogenic chicken DT40 cell lines with known DNA damage response pathways - wild type cell line |
| 743014 | qHTS assay for identifying genotoxic compounds that show differential cytotoxicity against isogenic chicken DT40 cell lines with known DNA damage response pathways - Rev3 mutant cell line |
| 743015 | qHTS assay for identifying genotoxic compounds that show differential cytotoxicity against isogenic chicken DT40 cell lines with known DNA damage response pathways - Rad54/Ku70 mutant cell line |
| 743053 | qHTS assay to identify small molecule agonists of the androgen receptor (AR) signaling pathway: Summary |
| 743054 | qHTS assay to identify small molecule antagonists of the androgen receptor (AR) signaling pathway using the MDA cell line: Summary |
| 743063 | qHTS assay to identify small molecule antagonists of the androgen receptor (AR) signaling pathway: Summary |
| 743067 | qHTS assay to identify small molecule antagonists of the thyroid receptor (TR) signaling pathway: Summary |
| 743077 | qHTS assay to identify small molecule agonists of the estrogen receptor alpha (ER-alpha) signaling pathway: Summary |
| 743078 | qHTS assay to identify small molecule antagonists of the estrogen receptor alpha (ER-alpha) signaling pathway: Summary |
| 743091 | qHTS assay to identify small molecule antagonists of the estrogen receptor alpha (ER-alpha) signaling pathway using the BG1 cell line: Summary |
| 743122 | qHTS assay to identify small molecule that activate the aryl hydrocarbon receptor (AhR) signaling pathway: Summary |
| 743139 | qHTS assay to identify aromatase inhibitors: Summary |
| 743140 | qHTS assay to identify small molecule agonists of the peroxisome proliferator-activated receptor gamma (PPARg) signaling pathway: Summary |
| 743199 | qHTS assay to identify small molecule antagonists of the peroxisome proliferator-activated receptor gamma (PPARg) signaling pathway: Summary |
| 743219 | qHTS assay for small molecule agonists of the antioxidant response element (ARE) signaling pathway: Summary |
| 743226 | qHTS assay to identify small molecule antagonists of the peroxisome proliferator-activated receptor delta (PPARd) signaling pathway: Summary |
| 743227 | qHTS assay to identify small molecule agonists of the peroxisome proliferator-activated receptor delta (PPARd) signaling pathway: Summary |
| 743228 | qHTS assay for small molecule activators of the heat shock response signaling pathway: Summary |
| 743239 | qHTS assay to identify small molecule agonists of the farnesoid-X-receptor (FXR) signaling pathway: Summary |
| 743240 | qHTS assay to identify small molecule antagonists of the farnesoid-X-receptor (FXR) signaling pathway: Summary |
| 743241 | qHTS assay to identify small molecule agonists of the vitamin D receptor (VDR) signaling pathway: Summary |
| 743242 | qHTS assay to identify small molecule antagonists of the vitamin D receptor (VDR) signaling pathway: Summary |

**Table S2.** Full list of “reactive, unstable, toxic” endpoint ToxAlerts found to be enriched in the binarized toxic set compared to the nontoxic set. See the ToxAlerts server (https://ochem.eu/alerts) for information on each alert.

| **Alert ID** | **Odds ratio** | ***p*-value** |
| --- | --- | --- |
| TA1000 | 35.23 | 4.69e-19 |
| TA975 | 27.62 | 9.60e-06 |
| TA567 | 25.06 | 3.09e-05 |
| TA1075 | 25.06 | 3.09e-05 |
| TA880 | 25.06 | 3.09e-05 |
| TA885 | 25.06 | 3.09e-05 |
| TA1089 | 16.36 | 4.99e-06 |
| TA914 | 16.36 | 4.99e-06 |
| TA998 | 16.36 | 4.99e-06 |
| TA520 | 13.8 | 4.61e-05 |
| TA972 | 8.17 | 6.15e-05 |
| TA877 | 8.17 | 6.15e-05 |
| TA561 | 6.73 | 2.34e-05 |
| TA871 | 6.44 | 5.66e-17 |
| TA1060 | 6.44 | 5.66e-17 |
| TA997 | 5.37 | 5.14e-05 |
| TA968 | 4.12 | 8.79e-10 |
| TA907 | 3.72 | 1.36e-04 |
| TA936 | 2.18 | 3.78e-06 |
| TA868 | 2.16 | 6.74e-06 |
| TA1079 | 1.84 | 5.76e-05 |
| TA1006 | 0.39 | 2.09e-06 |
| TA870 | 0.14 | 2.46e-05 |
| TA1022 | 0.1 | 1.13e-13 |
| TA887 | 0.09 | 4.71e-16 |
| TA1055 | 0.09 | 3.63e-12 |

**Table S3.** Protein target descriptors exhibiting a significant difference in distribution between binarized toxic set *vs.* nontoxic set. All targets which exhibited a significant difference and a positive value of Cohen’s *d* (indicating larger values among the toxic set) are included.

| **Uniprot** | **Cohen’s *d*** | ***p*-value** |
| --- | --- | --- |
| P00352 | 0.48 | 2.05e-17 |
| P51449 | 0.37 | 1.38e-13 |
| Q16236 | 0.36 | 5.54e-13 |
| Q8IUX4 | 0.33 | 1.49e-07 |
| P11473 | 0.28 | 1.03e-08 |
| Q12809 | 0.28 | 2.85e-05 |
| P10636 | 0.26 | 6.15e-06 |
| B2RXH2 | 0.25 | 3.63e-07 |
| Q96KQ7 | 0.24 | 1.33e-06 |
| P28335 | 0.24 | 1.52e-06 |
| O15296 | 0.24 | 5.99e-06 |
| P42858 | 0.23 | 5.21e-06 |
| P84022 | 0.23 | 5.19e-06 |
| P41595 | 0.22 | 1.20e-05 |
| O75496 | 0.21 | 2.09e-05 |
| P27338 | 0.2 | 3.84e-05 |
| Q9HC16 | 0.2 | 4.15e-05 |
| P28482 | 0.2 | 7.62e-05 |
| Q99720 | 0.19 | 8.31e-05 |
| O15118 | 0.18 | 2.07e-04 |
| P15428 | 0.18 | 2.56e-04 |
| P23975 | 0.18 | 3.15e-04 |
| P21397 | 0.18 | 3.15e-04 |
| P08913 | 0.18 | 3.22e-04 |
| P31645 | 0.18 | 3.77e-04 |
| O94782 | -0.18 | 2.12e-04 |
| Q96RI1 | -0.21 | 1.82e-05 |
| P08172 | -0.22 | 8.18e-06 |
| P34972 | -0.22 | 7.04e-06 |
| Q9UBN7 | -0.24 | 1.88e-06 |
| P11229 | -0.26 | 1.66e-07 |
| Q07869 | -0.3 | 2.35e-09 |
| P09917 | -0.31 | 9.43e-10 |
| P37231 | -0.32 | 1.16e-10 |
| Q9UQL6 | -0.34 | 7.43e-12 |
| P21554 | -0.35 | 2.24e-12 |
| P56524 | -0.39 | 7.41e-15 |
| P18031 | -0.45 | 7.06e-20 |

**Table S4.** Summary of compounds classified differently by models trained using molecular and target descriptors.

|  |  | **Test set** | | |
| --- | --- | --- | --- | --- |
| **Correct class of compound** | **Descriptors used by correct classifier** | **Random set %** | **Scaffold set %** | **Source set %** |
| Toxic | Molecular | 88.2 | 38.8 | 37.9 |
|  | Target | 11.8 | 61.2 | 62.1 |
| Nontoxic | Molecular | 60.6 | 78.3 | 74.7 |
|  | Target | 39.4 | 21.7 | 25.3 |

**Table S5.** Average and sample standard deviation of performances of models trained and tested using 20 different class-stratified random splits.

| **Classifier’s descriptors** | **Performance metric** | **Mean** | **Standard Deviation** |
| --- | --- | --- | --- |
| Molecular | ROC AUC | 0.914 | 0.013 |
|  | Average precision | 0.825 | 0.025 |
|  | CCR | 0.834 | 0.019 |
| Target | ROC AUC | 0.842 | 0.025 |
|  | Average precision | 0.719 | 0.041 |
|  | CCR | 0.761 | 0.027 |
| Tox21 assay | ROC AUC | 0.619 | 0.033 |
|  | Average precision | 0.403 | 0.034 |
|  | CCR | 0.598 | 0.029 |
| Molecular and target | ROC AUC | 0.913 | 0.013 |
|  | Average precision | 0.824 | 0.026 |
|  | CCR | 0.833 | 0.020 |

**Table S6.** Average and sample standard deviation of performances of models trained and tested using 20 different “single source” splits.

| **Classifier’s descriptors** | **Performance metric** | **Mean** | **Standard Deviation** |
| --- | --- | --- | --- |
| Molecular | ROC AUC | 0.851 | 0.013 |
|  | Average precision | 0.658 | 0.023 |
|  | CCR | 0.756 | 0.024 |
| Target | ROC AUC | 0.794 | 0.020 |
|  | Average precision | 0.626 | 0.033 |
|  | CCR | 0.708 | 0.020 |
| Tox21 assay | ROC AUC | 0.601 | 0.021 |
|  | Average precision | 0.375 | 0.022 |
|  | CCR | 0.575 | 0.023 |
| Molecular and target | ROC AUC | 0.852 | 0.013 |
|  | Average precision | 0.661 | 0.028 |
|  | CCR | 0.762 | 0.018 |
